# The low‐carbon risk society: Dilemmas of risk–risk tradeoffs in energy innovations, transitions, and climate policy

**DOI:** 10.1111/risa.14667

**Published:** 2024-06-29

**Authors:** Benjamin K. Sovacool

**Affiliations:** ^1^ Department of Earth and Environment Boston University Boston Massachusetts USA; ^2^ Institute for Global Sustainability Boston University Boston Massachusetts USA; ^3^ Department of Business Development and Technology Aarhus University Aarhus Denmark; ^4^ Science Policy Research Unit (SPRU) University of Sussex Business School Sussex UK

**Keywords:** climate mitigation, comparative risk analysis, energy security, low‐carbon transitions, risk tradeoffs

## Abstract

As countries and communities grapple with climate change, they seek to rapidly decarbonize their economies and cultures. A low‐carbon future will likely depend on more distributed solar energy, the electrification of mobility, and more efficient homes and buildings. But what emergent risks are evident within this low‐carbon society? This exploratory study first reviews the existing literature to identify 75 risk–risk tradeoffs by their category, medium of distribution, and type. It builds on these 75 examples to apply a typology of Risk Offsets, Risk Substitution, Risk Transfer, and Risk Transformation. Based on extensive document analysis, it applies that typology to three low‐carbon innovations: solar energy, battery electric vehicles, and building energy efficiency retrofits, identifying 36 distinct risk–risk tradeoffs in total. As such, the paper moves to discuss complexities and challenges in risk management. In doing so, it calls for a more refined risk assessment that better accounts for decision‐making considerations such as the magnitude or probability of risk, size of population exposed, certainty in risk estimation, severity of adverse outcome, distributional considerations, and the timing of risk impacts. It also summarizes emergent research gaps. Risk management in the context of climate action becomes a three‐dimensional chess game of weighing risk transmission, risk mediums, and risk categories.

## 1 INTRODUCTION

Modern society abounds with inherent risks. Almost everything humans do engenders some degree of risk—a short drive for a dinner out brings small and large dangers including those of automobile crashes, poisoned or spoiled food, slipping and falling in the restaurant, or being subject to a robbery at the cash register. So too with the decision to cycle or walk or even to stay at home. If you play basketball to improve your health, you can endanger your health with the risk of injuring your body. If you take an aspirin to treat a headache, it may come with a countervailing risk of a stomachache. As Graham and Wiener (1995, p. 9) wrote about three decades ago, in contemporary times “whenever you reduce or eliminate one risk, you may simultaneously increase or create another.”

This creates a puzzle for modern risk management, given that emergent technologies or practices tend not to eliminate or reduce risk but to redistribute risk. Luhmann (1993) cautioned that highly industrialized and technologically sophisticated societies tend to obscure the social construction and distribution of risk, given that a multiplicity of interactions makes causal attribution difficult, and because of a time lag between when technologies are adopted and when their consequences become clearer. Beck (1992) added that a hallmark of industrial times is that it has become based profoundly on the production and distribution of risk types and exposures, creating what he famously termed a “risk society.”

But what emergent risks are evident within a low‐carbon society? As countries and communities grapple with the challenge of climate change and seek to rapidly decarbonize their economies and cultures, global society is set to undergo rapid and deep transitions to renewable energy, cleaner forms of mobility, and more efficient and climate‐friendly homes and buildings. For instance, the International Energy Agency (2023a) reports that the installed capacity of solar power will become the largest single source of energy, surpassing that of coal, by 2027, with a consequent doubling in growth rate every 1–3 years. Battery electric vehicles (EVs) are poised for similar ascension, with the International Energy Agency (2023b) projecting that they will reach 65% of all car sales by 2030, will achieve average annual growth rates of 25% between now and then, and will reach 1 billion adopters by 2040. Energy efficiency efforts are also playing a prominent role in climate and energy policy, with 46 large countries committing to a doubling in the rate of efficiency progress and estimations that efficiency will further reduce energy bills in advanced countries by one‐third and make up 50% of carbon dioxide reductions by 2030 (International Energy Agency, 2023c). That all three of these transitions—solar energy, EVs, household efficiency—are urgently needed to address the risks of climate change is evident in the latest report from the Intergovernmental Panel on Climate Change (IPCC, 2023), the Fifth National Climate Assessment in the United States (Jay et al., 2023), and a systematic review of 100,000 peer‐reviewed studies on climate action (Creutzig et al., 2021).

Nevertheless, even these low‐carbon innovations have intrinsic risks. This exploratory study examines those risks with solar energy, battery EVs, and energy efficiency retrofits by utilizing a typology of risk–risk tradeoffs, confirmed by 75 purposive examples, and consisting of Risk Offsets, Substitution, Transfer, and Transformation. It then applies that typology to the three low‐carbon innovations, showing evidence from document analysis of how each innovation perpetuates *all four* types of tradeoffs. It last discusses challenges in risk management and introduces a framework for resolving, or at least weighing, risk–risk tradeoffs. Such an analysis is needed not only to promote a clearer comprehension of the risks, costs, and benefits with decarbonization but also to focus on more cost‐effective policy interventions and efforts at risk reduction (Congressional Research Service, 2011). As economist Esty (1996, p. 12) has written, such an analysis of risk–risk tradeoffs serves as a basis for sound policymaking.

## 2 CASE STUDY SELECTION, RESEARCH DESIGN, AND LIMITATIONS

This section briefly introduces our case study selection of three low‐carbon innovations before it details our research design of three consecutive literature reviews. It ends by discussing limitations with our approach.

### 2.1 Case study selection of three low‐carbon innovations

Our first low‐carbon innovation selected is community solar energy. Around the world, cities, companies, governments, and households are coming to embrace community and household or residential solar energy as a core part of a shift toward sustainability, decentralization, community ownership, and enhanced control over energy supply (Allen et al., 2019; Brisbois, 2019). Solar panels have seen costs fall dramatically over the past two decades, generate electricity without any combustion, can be installed modularly on almost any home or roof, and tend to have levelized costs of energy among the most competitive in the electricity market. Shidore and Busby (2019) frame solar energy as a vital part of a pathway to become more energy secure nationally as well as a mechanism to address energy scarcity and poverty. Dolter and Boucher (2018) discuss solar energy as an instrumental part of achieving energy justice.

More recent projections of future growth and investment are startling. Stanbery et al. (2023) conclude that an “unprecedented ramp‐up of production capacity” over the next two decades is expected to occur, with solar installations growing from 1000 gigawatts to 63.4 terawatts by mid‐century, or a *60‐fold increase* from today. The Solar Energy Industries Association (2023) has translated what these trends mean for the United States, and it expects that the Inflation Reduction Act alone will drive $565 billion in new solar investment over the next decade. This trend holds true globally as well, with the International Energy Agency (2023d) now forecasting that more investment went into the solar energy sector in 2024 than to the production of oil. Looking forward, the International Energy Agency (2023d) reports that low‐emissions technologies are expected to account for almost 90% of investment in electricity into the future and that for every dollar invested in fossil fuels, about $1.7 are now going into clean energy sources such as solar power.

Our second innovation selected is battery EVs. Globally, the transportation sector continues to rely predominantly on fossil fuel energy in the form of crude oil and gasoline, with serious adverse consequences for climate change, air pollution, and other negative social impacts. Many researchers, policymakers, and other stakeholders view a widespread transition to EVs as both feasible and socially desirable (Tran et al., 2012; Williams et al., 2012), given that such vehicles can reduce carbon dioxide emissions, compared to conventional cars by 40%–60% (Addison et al., 2010; Kutscher, 2007). Battery EVs can also enhance sustainability outcomes when they are coupled to ridesharing, public transportation, or automation (Adler et al., 2019) and/or are powered and recharged by low‐carbon electricity sources. EVs—in both full battery configuration or as hybrids—can consequently address the dual target risks of climate change and inefficient and less affordable transportation.

Global investment trends for EVs are similarly astonishing. The International Renewable Energy Agency (2018) estimates that the number of EVs will rise almost *1000‐fold* by mid‐century, expanding from about 1 million cars in 2015 to about 1 billion passenger cars by 2050. The International Energy Agency (2024) adds that sales of new EVs keep rising and will surpass 17 million in 2024, accounting for more than one in five cars sold worldwide and that about $500 billion is already invested annually in EV and battery manufacturing facilities. The International Energy Agency (2024) also tracked more than 20 major car manufacturers, representing more than 90% of global car sales in 2023, that have set electrification targets.

Our third is household energy efficiency retrofits. Energy efficiency retrofits focus on redesigning homes, especially space heating (in Northern countries) or cooling (in tropical countries), to reduce their energy demand. This is typically achieved through the integration of multiple technologies including fabric insulation, heat pumps and mechanical ventilation heat recovery, energy‐efficient lighting, energy‐efficient appliances, improved windows, and even integration with solar panels (Jones et al., 2017). The advantages of energy efficacy retrofits encompass environmental benefits such as reduced carbon emissions, medical benefits such as improved occupant health, economic benefits such as revitalized local enterprises, and equity benefits such as reduced rates of energy poverty (Kerr et al., 2017). Retrofits can also improve property values, enhance thermal comfort, and result in major savings on energy bills (Brown et al., 2019).

Although it is more difficult to discern precise numbers given the distributed nature of energy efficiency investments, the International Energy Agency (2023e) reports that in response to the energy crisis of 2022, countries representing 70% of global energy demand have introduced or strengthened efficiency policy packages and that annual energy efficiency investment is up 45% since 2020, with about $700 billion being spent on energy efficiency investment support. The Inflation Reduction Act of 2022 in the United States by itself included $86 billion in support for energy efficiency actions. Grand View Research (2022) offers a more nuanced assessment of the size of the energy retrofit market in the United States—inclusive of building envelopes, appliance upgrades, lighting replacements, and improvements to heating, ventilation, and air condition systems—estimating a value of about $150 billion that year.

### 2.2 Research design

To capture and elaborate on risk–risk tradeoffs on these three innovations, this study combines an “umbrella review” and a “critical review” (Grant & Booth, 2009). An umbrella review refers to the compiling of evidence from multiple disciplines or literature searches into one accessible and usable document, highlighting (at times) competing interpretations and uncertainties in the literature. A critical review requires both a sufficient review of existing literature and a degree of analysis, interpretation, and conceptual innovation. The critical aspect of our review enables us to describe and interpret what is known, whereas the umbrella aspect of our review enables us to capture diverse interdisciplinary perspectives and identify recommendations for research.

To provide more detail, three specific interdisciplinary desk‐based literature reviews and document analyses were undertaken. The first was of the literature on definitions and conceptions of risk, with an emphasis on categories of risk, mediums of risk distribution and transfer, and theories of risk–risk tradeoffs. The second literature review was equally targeted, and it involved searching the literature for explicit studies offering empirical examples of risk–risk tradeoffs. This second review was grounded mostly in risk governance literature, particularly literature on risk pre‐assessment, including risk identification. The third and final literature review was more broad, and it focused explicitly on the types of risk–risk tradeoffs inherent in the three low‐carbon innovations of community solar energy, EVs, and household efficiency retrofits.

All three literature reviews involved both Scopus (to capture the academic literature) and Google Scholar (to capture books or any relevant reports, including those from the National Academies or other academic groups, and any relevant gray literature). All three reviews also focused on relevant literature published over the past 20 years (from 2005 to 2024), and all reviews examined only studies in English.

### 2.3 Limitations

In laying out this research design and line of argumentation, limitations deserve to be mentioned. First, most generally the idea of risk–risk tradeoffs in the domain of climate change or health has compared the risks of acting to do something, for example, addressing climate change (via mitigation efforts such as those in this paper, e.g., EVs, community solar energy, and energy efficiency), to the risks of not doing anything, for example, greater risks of climate change through inaction. In this way, this paper focuses on only half of the existential risk–risk dilemma of acting to implement climate mitigation efforts, not the continuing risks of failing to implement them, which would mean greater reliance on fossil fuels, and greater climate change hazards.

Second, this paper focuses only on the negative risk–risk tradeoffs engendered through solar energy, EVs, and efficiency retrofits. It does not focus on some of the positive co‐benefits to their adoption including greater household resilience to blackouts (a benefit of solar, see Hasselqvist et al., 2022), better built and longer‐lasting vehicles with far lower carbon footprints than conventional cars (a benefit of most EV brands, see Archsmith et al., 2015; Ellingsen et al., 2016), or the health improvements that retrofits can bring to building occupants (see Tieskens et al., 2021). In this way, again, the paper only brings into focus half of the risk dimensions for each low‐carbon innovation.

## 3 CONCEPTUALIZING RISK AND VALIDATING A TYPOLOGY OF RISK–RISK TRADEOFFS

To begin, this section of the study defines “risk” and “risk–risk tradeoffs” before discussing categories of social risk, mediums of risk distribution, and types of risk transfer. It then utilizes 75 examples of risk–risk tradeoffs published in the literature to validate the typology that will then be applied to the three low‐carbon innovations in Section 3.

### 3.1 Categorizing risk and defining risk–risk tradeoffs

Rather than advancing new definitions and conceptions of risk, this study relies on well‐established definitions. Most broadly, risk can be defined as “the chance of an adverse outcome to human health, the quality of life, or the quality of the environment” (Graham & Wiener, 1995, p. 12). Risk can therefore reflect any or all of the five conceptions of risk defined by Hansson (1989, 2016) and Möller et al. (2006), that is, risk can reflect (1) an unwanted event that may or may not occur, (2) the cause of an unwanted event that may or may not occur, (3) the probability of an unwanted event that may or may not occur, (4) the statistical expectation value of unwanted events that may or may not occur as well as, and (5) the fact that a decision is made under conditions of known probabilities (“decision under risk”). In simpler terms, and in the context of climate change and climate action, risk can also be envisioned as a function of hazards and vulnerability and exposure to them (Šedová et al., 2024).

These risks, hazards, and exposure to them can also cut across different dimensions or categories in society. Lupu (2019) notes how natural risks may involve phenomena such as earthquakes or forest fires, whereas technological risks can involve industrial pollution and chemical spills (see Table 1). These risks are categorically distinct however from biological risks (affecting nature or the natural environment), which may involve epizootic diseases or the spread of pests, and differ still from economic risks such as financial crises, demographic risks such as aging, political risks such as trade disputes, or medical risks such as the incidence of disease (affecting public health and human beings). Such dimensions confirm the presence of “systemic risk” across risk categories as well—hazards that are complex, uncertain, ambiguous, and have the potential to reverberate throughout political, social, and economic dimensions (Klinke & Ortwin, 2006; Sidortsov, 2014).

**TABLE 1:** **TABLE** 1 Seven categories and examples of social risk.

Categories	Examples
Natural	Earthquakes, landslides, volcanic eruptions, storms, floods, tornadoes, aridity and drought, avalanches, frost, forest fires, hail, and heavy rainfall
Technological	Industrial pollution, industrial accidents, nuclear radiations, toxic wastes, dam failures, transportation hazards, plant explosions, fires, chemical spills, violations of privacy, cyberattacks, and hacking
Biological	Epidemics, epizootic diseases, genetic contamination, spread of pests, and exposure to natural toxins
Economic	Changes in supply or demand, economic shocks, financial crisis, labor market instability, unemployment, and regulation
Demographic	Alterations in household dynamics, population aging, demographic dependency, racism, sexism, colonialism, crime, culture, or degradation of social status
Political	Activism and protest, political transformations, wars, sanctions, and trade disputes
Medical	Disease, death, accidents, injury, morbidity, mortality

*Source*: Modified from Lupu (2019).

Risk–risk tradeoffs are defined as “the change in the portfolio of risks that occurs when a countervailing risk is generated (knowingly or inadvertently) by an intervention to reduce the target risk” (Graham & Wiener, 1995, p. 23). They are also sometimes known as “side effects” or “collateral damage” (Lofstedt & Schlag, 2017), “risk tradeoffs” (Gayer et al., 2000), “interacting and interconnected risk” (Pescaroli & Alexander, 2018), and “burden shifting” (Kolcava et al., 2019) or “problem shifting” (Hansen & van den Bergh, 2024). In even simpler terms, risk–risk tradeoffs refer to when new risks are created while addressing existing ones (Hansen et al., 2008). Oftentimes, efforts to solve one target risk can lead to new, unintended, or even more dangerous risks that increase net risk to society rather than reducing it. They can also involve tradeoffs within and between the seven categories of risk. For example, efforts to mitigate the natural risk of storm surge could be to erect seawalls and dykes, but these (if built from wood) could be vulnerable to forest fires or landslides. Or efforts to address the biological risk of future epizootic disease outbreaks by inoculating animals or plants could increase the economic risk of goods and services being too expensive for consumers to purchase.

### 3.2 Mediums of risk distribution

Risk–risk tradeoffs have mechanisms of dissemination. When in the form of damage to the environment, Hahn and Males (1990, p. 26) classically refer to these as “mediums of pollution,” as they refer to a specific “target medium” such as air, water, or land, but also a “shifted medium” where the risk gets morphed into a new dimension. These are sometimes called “cross‐media shifts” to reflect the movement of pollution or risk from one medium to another. Such shifts can be the direct result of regulation or the indirect result of voluntary behavior. Examples include air pollution efforts where scrubber water is discharged into streams—this shifts the medium of pollution from air to water. Such multi‐media shifts can be complex, as one example the Environmental Protection Agency (1992) noted no less than 14 pathways for shifting mediums concerning groundwater and sewage sludge alone.

**FIGURE 1:**
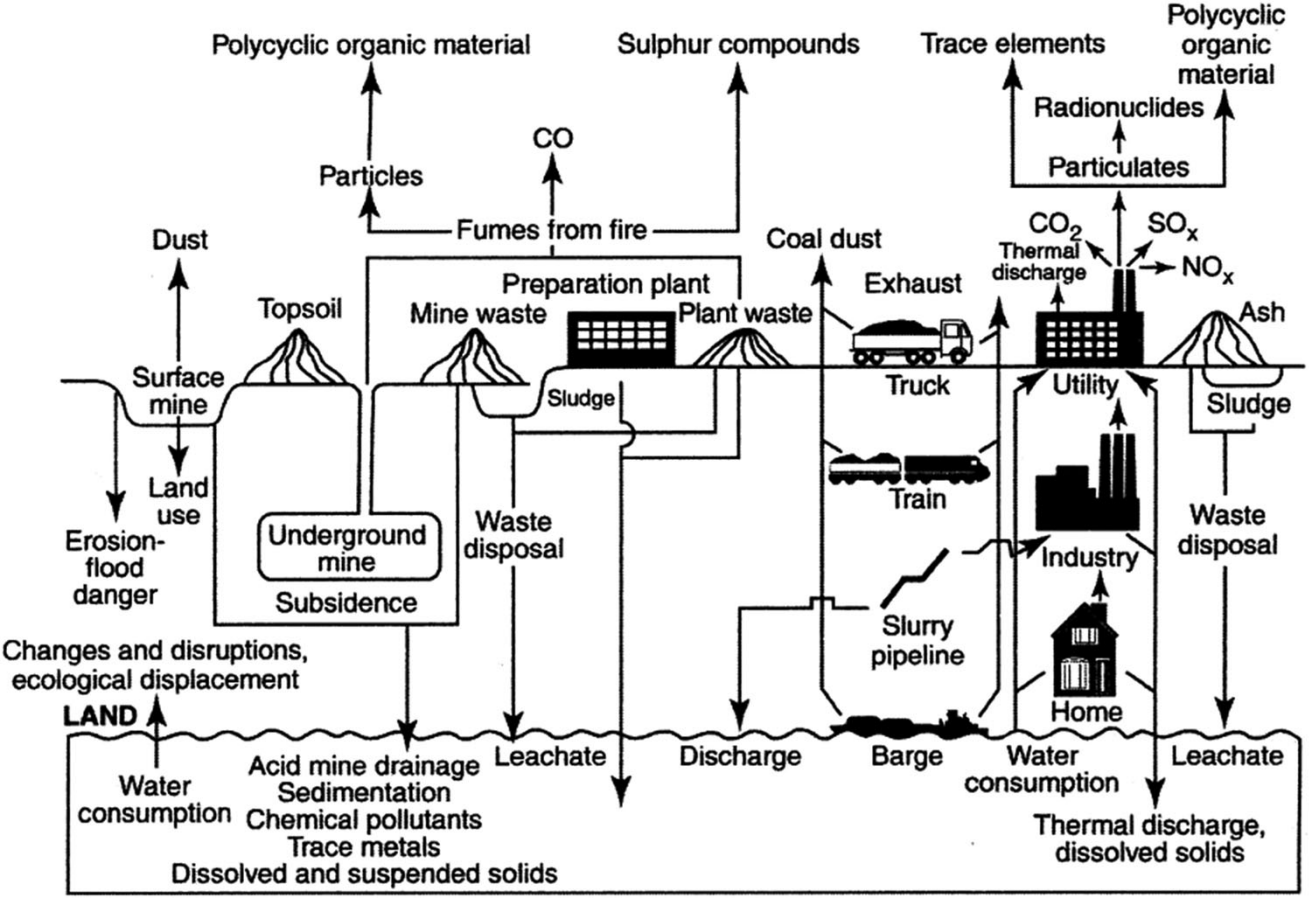
The multiple mediums of risk distribution with coal‐fired electricity generation. *Source*: Drawn from the US Department of Energy and the Commission of the European Communities (1992, A1–A3), used with permission.

Graham and Wiener (1995) argue that a focus on mediums of pollution is even built into how institutions are structured to reduce environmental risks. The Environmental Protection Agency in the United States is internally subspecialized and structured into medium‐specific subagencies: air, radiation, water, pesticides, toxic substances, and wastes. Adapting this work, one can envision eight specific mediums of risk transfer relevant for low‐carbon innovations: (1) air, including stationary and mobile sources; (2) radiation or infection, including stress, deteriorated physical, or mental health; (3) water, including drinking water, freshwater, and the oceans; (4) pesticides; (5) toxic substances; (6) wastes, including solid and hazardous wastes; (7); land, including forests and habitats; and (8) technology‐driven mediums, for example, where a device such as airbags, automated cars, or guns directly cause a risk themselves. Sometimes, as Figure 1 indicates, a single activity such as combusting coal in a power plant can produce multiple risks and adverse outcomes among multiple mediums simultaneously.

### 3.3 Types of risk transfer

A final attribute important for the analysis of risk–risk tradeoffs are the types of risk transfer. Hornstein (1992) even refers to this process as the cornerstone of “comparative risk analysis.” Graham and Wiener (1995) offer a robust typology suited to this task. For them, a “target risk” is the risk that is the primary focus of a risk reduction effort or policy goal. In the example of hypertension, the target risk would be the chance of a heart attack or stroke due to high blood pressure. The “target population” is the group of individuals who hope to benefit from the amelioration in the target risk. The “countervailing risk” is the chance of an adverse outcome that results from any attempt to reduce the target risk. Sticking with the example of hypertension, a countervailing risk could be the side effects of heart medication or the risk of death from anesthesia during heart surgery. Moreover, risks can be transferred not only within a target population but to other populations as well, say exposure to toxic substances or accidents on the floor of the factory where the workers are manufacturing the heart medication or the risk that the surgeon performing heart surgery has a traffic accident en route to the hospital.

**FIGURE 2:**
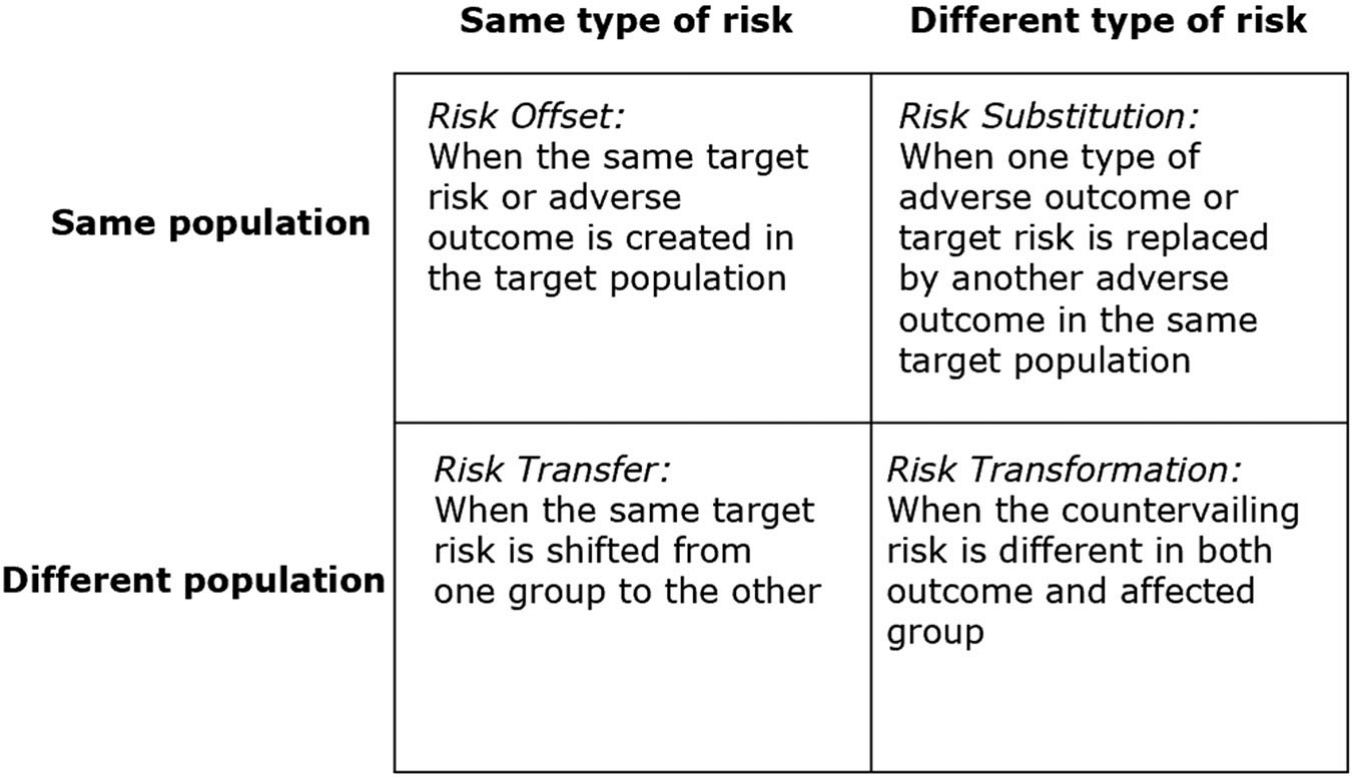
A typology of risk–risk tradeoffs. *Source*: Adapted from Graham and Wiener (1995) and Rascoff and Revesz (2002).

As shown in Figure 2, this leads to four analytical categories of risk–risk tradeoffs: Risk Offset, Risk Substitution, Risk Transfer, and Risk Transformation. Where a countervailing risk affects the same target risk for the same population, this is a Risk Offset. Where a countervailing risk involves the same target risk but shifts it to a different population, it is a Risk Substitution. When a countervailing risk involves a different target risk but the same population, this is a Risk Transfer. And when a countervailing risk involves both a different risk and a different population, this is a Risk Transformation.

Some examples can drive home the utility and explanatory power of his typology. In the upper left of Figure 2, we have Risk Offset, where the same target risk occurs in the same target population. An example would be banning fungicides on foods because they create a target risk of cancer, but this frees up fungi to spread other cancerous compounds into foods. In the upper right is Risk Substitution, where a new risk replaces the target risk in the same population—this could be where banning fungicides on foods causes the price of organic food to go up, stimulating consumers to eat cheaper, less healthy, salty foods that contribute to cholesterol—the target population reduces the risk of chemical exposure but increases the risk of heart disease. In the bottom left of Figure 2, we have Risk Transfers, when the same risky outcome is shifted from one population to another. An example would be reducing toxic air emissions from an industrial facility only by generating hazardous wastes that are sent to a landfill—the risks of adverse outcomes from chemical exposure are merely shifted from one population (those breathing air near the facility) to another (those near the landfill). And finally, in the bottom right we have Risk Transformation, a new risk reaching a new population, such as closing that polluting industrial facility to protect the air of those living nearby, which then leads to a new risk of job losses and unemployment among a new group, that is the facility's workers.

Distinguishing among these various risk–risk tradeoffs in a typology of target populations, other populations, target risks, and countervailing risks is beneficial for at least three reasons. It draws attention to an important attribute of risk analysis, how direct or immediate a relationship exists between an action to reduce risk and a potential ancillary effect. It reveals where risk–risk tradeoffs may cluster analytically into families and continuities. Last, it avoids terminological confusion and suggests a coherent approach to analyzing tradeoffs.

### 3.4 Analyzing 75 risk–risk tradeoffs

The categories of risk, mediums of risk distribution, and types of risk transfer offer an effective framework for comparative risk analysis. To illustrate this, 75 examples of risk–risk tradeoffs were identified in the literature. To be clear, this is not an exhaustive or representative list, just an illustrative one based on purposive sampling as the author read the literature to provide a diverse range of examples and types. Nevertheless, it involves the following 75 examples, with more details for each example (its description, target risk, countervailing risk, category of risk, medium of risk transfer, and source for the claim) offered in the Appendix. Presenting such a wide collection of risk–risk tradeoffs shows how they occur across very different products, actions, or regulations and indeed involve different industries and sectors and different actors at different levels ranging from individuals to companies and governments. The 75 examples are, in alphabetical order:

1. 1977 Clean Air Act
2. Acid rain abatement
3. Airbags
4. Antidepressant drug treatment
5. Artificial sweeteners
6. Automated vehicle adoption
7. Avoiding eating fish
8. Ban of antibiotic growth promoters in animal feed
9. Ban on chlorofluorocarbons (CFCs)
10. Banning ethylene dibromide
11. Banning leaded paint in buildings
12. Banning nitrates in food
13. Banning organophosphates/carbamate pesticides
14. Banning the pesticide Alar (daminozide)
15. Bans of chlorinated solvents in the vehicle repair industry
16. Bans on asbestos in automobiles
17. Bans on asbestos in buildings
18. Bicycle lanes
19. Bioenergy with carbon capture and storage
20. Breastfeeding babies
21. Chemotherapy
22. Child safety caps on medicine bottles
23. Chlorinated drinking water
24. Clean coal technologies
25. Clozapine therapy
26. Coral reef protection in Australia
27. Delaney Clause
28. Diapers
29. Domestic bans of dichlorodiphenyltrichloroethane (DDT)
30. Enhanced weathering
31. Estrogen therapy
32. Fuel economy standards for vehicles
33. Fuel‐substitution of coal with natural gas
34. Genetically modified food in Zambia
35. Global bans of DDT
36. Hazardous waste cleanup
37. Health and climate impacts of carbon capture and direct air capture
38. Heath risks of hospitalization
39. Hormone replacement therapy
40. Lead battery recycling
41. Legalizing drugs, alcohol, or prostitution
42. Light rail mass transit
43. Mandatory minimum legal sentencing
44. Mass inoculation against parasitic worms
45. Mobile phones
46. Monuments in Washington DC
47. Ocean dumping of industrial wastes
48. Ownership of firearms
49. Prohibitions of drugs, alcohol, and prostitution
50. Public participation against pipelines
51. Public participation against shale gas in the United Kingdom
52. Radiation therapy for prostate cancer
53. Recycling plastic water bottles
54. Reducing ground‐level ozone
55. Reformulated gasoline
56. Remediation of the ExxonMobil Valdez oil spill
57. Replacing phosphorus in detergents with nitrilotriacetic acid
58. Restrictions on elderly drivers
59. Ridesharing and carpooling
60. Seat belts
61. Stratospheric aerosol injection
62. Substituting coal‐fired electricity with nuclear power
63. Substituting leaded gasoline with methyl tert‐butyl ether
64. Substituting nuclear power with coal‐fired electricity
65. Substitution of ammonia, methyl chloride, and sulfur dioxide with CFCs
66. Sustainable forest management
67. Synthetic chemical pesticides
68. Tamoxifen
69. Tube well drilling in South Asia
70. Ultraviolet sunscreen
71. Use of bromopropane in cleaning solvents
72. Use of hydrochlorofluorocarbon as an alternative to CFCs
73. Vegetarian and pescatarian diets
74. West Nile Virus control
75. Wind power and sulfur hexafluoride (SF6)

**FIGURE 3:**
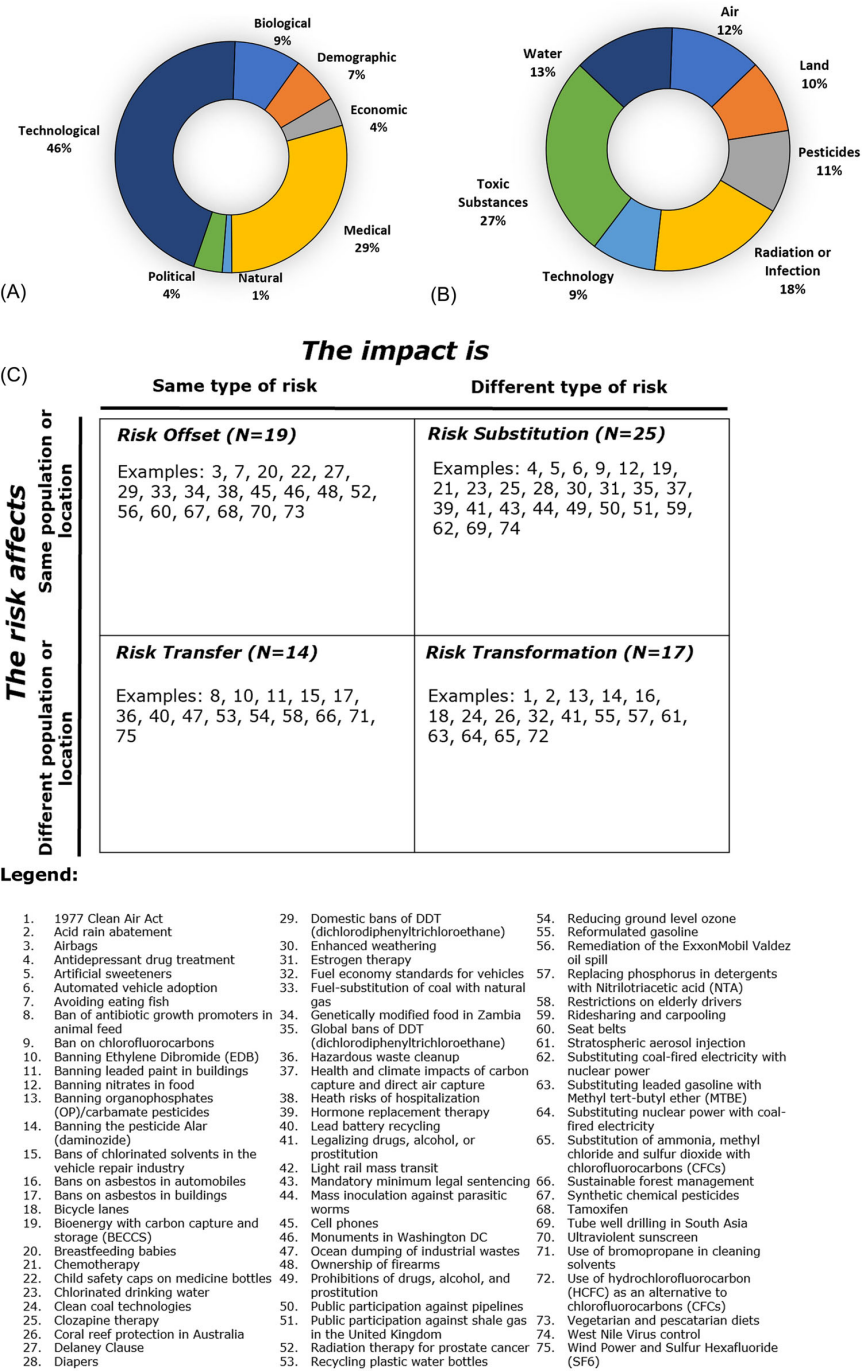
Validating risk categories (A), risk mediums (B), and the typology of risk–risk tradeoffs (C) among 75 examples. *Source*: Author.

Three trends become apparent with assessing this corpus of 75 risk–risk tradeoffs. First, Figure 3A indicates, when coded mutually exclusively (i.e., a particular risk–risk tradeoff had only one primary categorization), the largest single category of risk is technological followed by medical and biological. Natural, political, and economic risks were less common within this evidence base.

Second, risk mediums could be hybrid and were thus coded to be inclusive rather than mutually exclusive (a single risk–risk tradeoff could have multiple mediums as the Appendix also shows). When analyzed in this fashion, Figure 3B shows how the most common medium of risk is toxic substances, followed by radiation or infection, water, and air. Less common mediums were technology, pesticides, and land. This is interesting, as it suggests an inverse relationship between risk categories and mediums—that is, the categories of technological and medical risk dominate the evidence base but seem to involve non‐technological and medical mediums such as toxic substances, water, and air.

Third and last, the 75 examples fit neatly into and validate the typology, with 19 examples of Risk Offset, 25 examples of Risk Substitution, 14 examples of Risk Transfer, and 17 examples of Risk Transformation apparent. Some of these are direct tradeoffs, such as Example 73, Eating more Fish (risk of toxic substances), or Example 7, Avoiding Eating Fish (risk of infection); Example 28 of using cloth diapers (risk of air pollution for washing) or disposable diapers (risk of waste); or Example 49 banning alcohol or drugs (risk of crime) or Example 41 legalizing alcohol or drugs (risk of addiction). Others are coupled risks, such as Example 62, phasing out coal‐fired power for nuclear power (the risk of nuclear accidents) coupling to Example 64 (phasing out nuclear power in favor of coal increases the risk of coal mining accidents).

## 4 RESULTS: RISK–RISK TRADEOFFS AND LOW‐CARBON INNOVATIONS

In examining the 75 examples in Section 2, three shortcomings also become apparent. Most of the examples relate generally to the environment or to medical treatment and health; specific applications to energy and climate policy or low‐carbon innovations are infrequent. Most examples involve only a small number of categories of risk and generally one primary medium or at most two mediums. Finally, all the examples fit neatly into *one* quadrant of the typology.

These shortcomings point the way toward a more integrated approach that focuses on low‐carbon innovations, which focus on multiple categories of risk, across multiple mediums (perhaps as many as all of them), in ways that may see risks multiply across *all* of the quadrants of the typology simultaneously. This is precisely the case with Section 3, which reveals how solar energy, EVs, and energy efficiency retrofits involve risk–risk tradeoffs across a manifold number of risk categories and mediums and across the entire typology of Risk Offsets, Risk Substitution, Risk Transfer, and Risk Transformation. It does so by illustrating 12 specific risk–risk tradeoffs for each innovation, involving all aspects of the typology, also based on a review of the published literature.

### 4.1 Community solar energy

**FIGURE 4:**
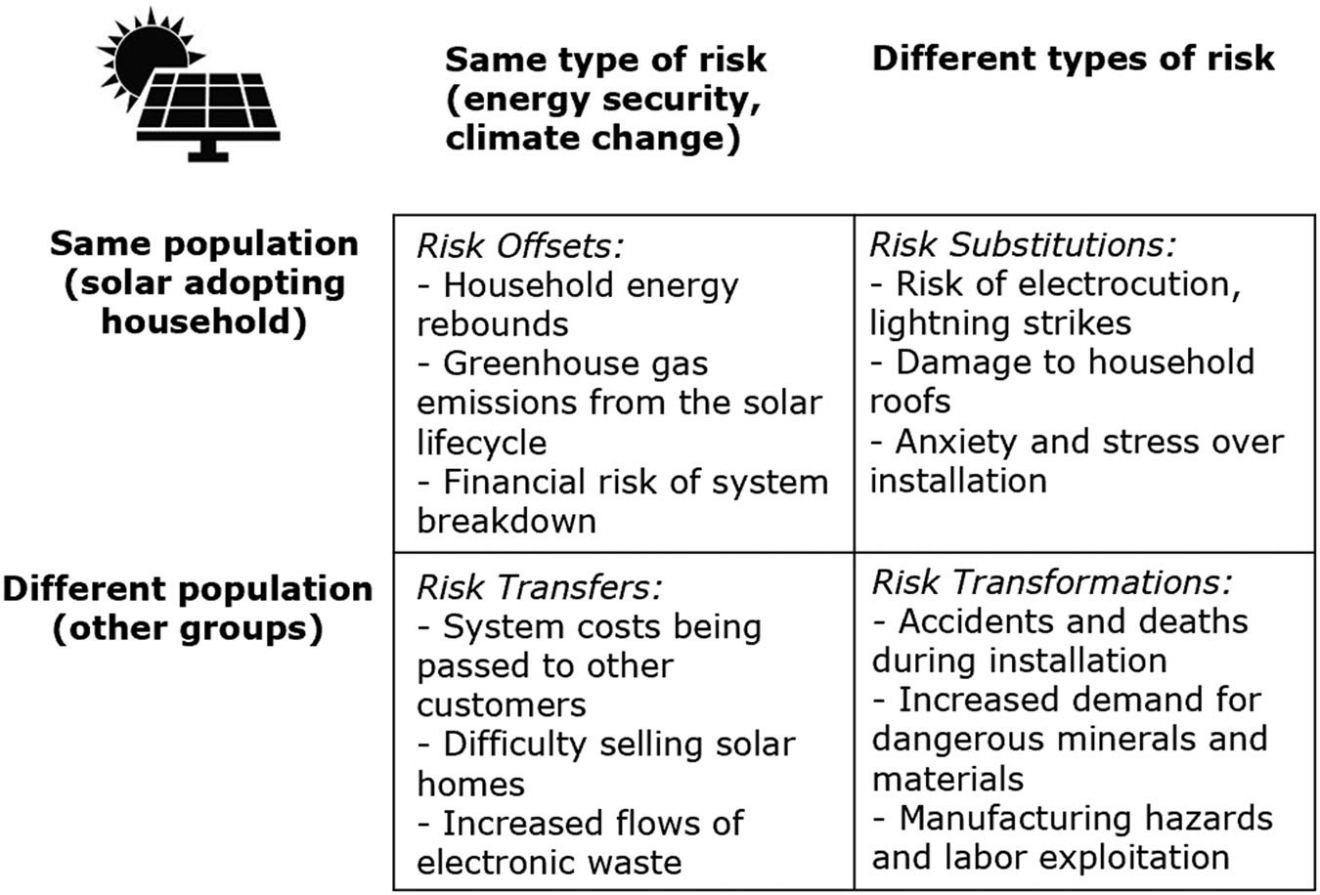
Depicting 12 risk–risk tradeoffs with household solar energy. *Source*: Author.

Community and household solar energy is intended to reduce two target risks simultaneously: climate change and household energy insecurity. However, evidence does exist that solar energy adoption involves Risk Offsets that aggravate these goals with countervailing risks (see Figure 4). One of these is household energy rebounds or large increases in energy consumption post adopting a solar panel. This can be direct, whereby households feel morally licensed to consume more electricity given they have “done their part” to help the environment, or indirect, whereby households utilize their financial savings from solar adoption to purchase other environmentally damaging things like vacations, hot tubs, video game systems, or cars (Beppler et al., 2021; Galvin et al., 2022; Sovacool et al., 2022). Greenhouse gas emissions also occur across the solar energy lifecycle, including emissions of carbon dioxide and methane with the manufacturing process, as well as the consumption of plastics, petroleum, and natural gas used for heating and processing along with hazardous materials such as cadmium (Mulvaney, 2013; Nugent & Sovacool, 2014). The manufacturing of thin‐film solar photovoltaic panels relies on sulfur hexafluoride and nitrogen trifluoride as etching gases, both very potent trace gases with extremely high global warming potential (Becken et al., 2011). A final tradeoff is the financial risk of a system breakdown. When batteries, panels, or inverters fail, solar panels can become a net financial burden for adopters rather than an asset (Sovacool et al., 2022).

As Figure 4 indicates, Risk Substitutions occur as well. Solar panels need regularly cleaned to operate optimally, and thus expose household owners to the risk of electrocution and even death (Williams, 2021) or being struck by lightning when on rooftops. Installation, especially when done improperly or poorly, can contribute to permanent roof damage as well as alterations to the color and appearance of roofs, in addition to leaks (Sovacool et al., 2022). Some owners of solar panels have also reported increased stress, depression, and anxiety before and during installation, especially among widows or widowers who saw the installation process as traumatic because it forced them to clean out attics in the United Kingdom (Sovacool et al., 2022). Ransan‐Cooper et al. (2020, p. 5) noted that some Australian homes adopting solar panels were frustrated when they “could not understand and control the technology in the way desired and in line with islander attitudes of self‐reliance and autonomy.”

Solar is prone to Risk Transfers. Energy suppliers and electric utilities often spread the cost of upgrading the transmission and distribution system to all households, not just solar adopters (Strielkowski et al., 2017). Solar energy adoption patterns are strongly mediated by race and class, with disparities in adoption among communities of color (Sunter et al, 2019). Real estate agents report that it can be more difficult to sell homes with solar panels on them, given that buyers may be more hesitant if they do not understand the benefits or have concerns about maintenance and repairs, they may feel unqualified to discuss solar, or they may worry solar will scare away potential buyers (Palmetto, 2022). Solar systems also generate their own flows of waste and electronic waste (Atasu et al. 2021; Cucchiella et al., 2015; IRENA & IEA‐PVPS, 2016), which can contribute to climate change via greenhouse gas, black carbon, and particulate matter emissions.

Finally, Risk Transformations transpire. Accidents and deaths can occur during installation, with the US Bureau of Labor's National Census of Fatal Occupational Injuries showing more than 100 fatal injuries a year among rooftop solar installations, a number that increases as much as 15% per year as the volume of solar installations grows (Wang, 2021). Surging demand for solar has a concomitant increase in the need for energy transition metals such as copper, cobalt, lithium, and rare earth minerals, which are environmentally damaging to produce and can result in community displacement and disruption in mining towns (Svobodova et al., 2022), including the risk of subsidence and earthquakes. Solar energy last involves manufacturing hazards, given that heavy metals such as lead, tin, and cadmium are hazardous for workers (Mulvaney, 2014; Salim et al., 2019). Solar manufacturing connects with patterns of labor exploitation, including poor working conditions or unfair treatment of solar employees (Brock et al., 2021; Stock, 2021), which can even lead to political risks such as populism, protest, and failed industrial strategy implementation, which happened in Germany. Murphy and Elimä (2021) document the use of forced labor within the solar supply chain in the Uyghur Region of China where polysilicon manufacturers and quartz suppliers expose workers to hazardous conditions and pesticides and rely on forced labor transfers or are located within industrial parks where labor transfers are common. Stock and Sovacool (2023, 2024) similarly document occupational hazards connected to quartz mining and the silica industry in India, a sector they note is prone to weak governance and rampant corruption, with much production controlled by “sand mafias” with illegal lease claims that bribe politicians, government agencies, and private industries to ignore or enable the social and environmentally destructive mining practices

### 4.2 EVs

**FIGURE 5:**
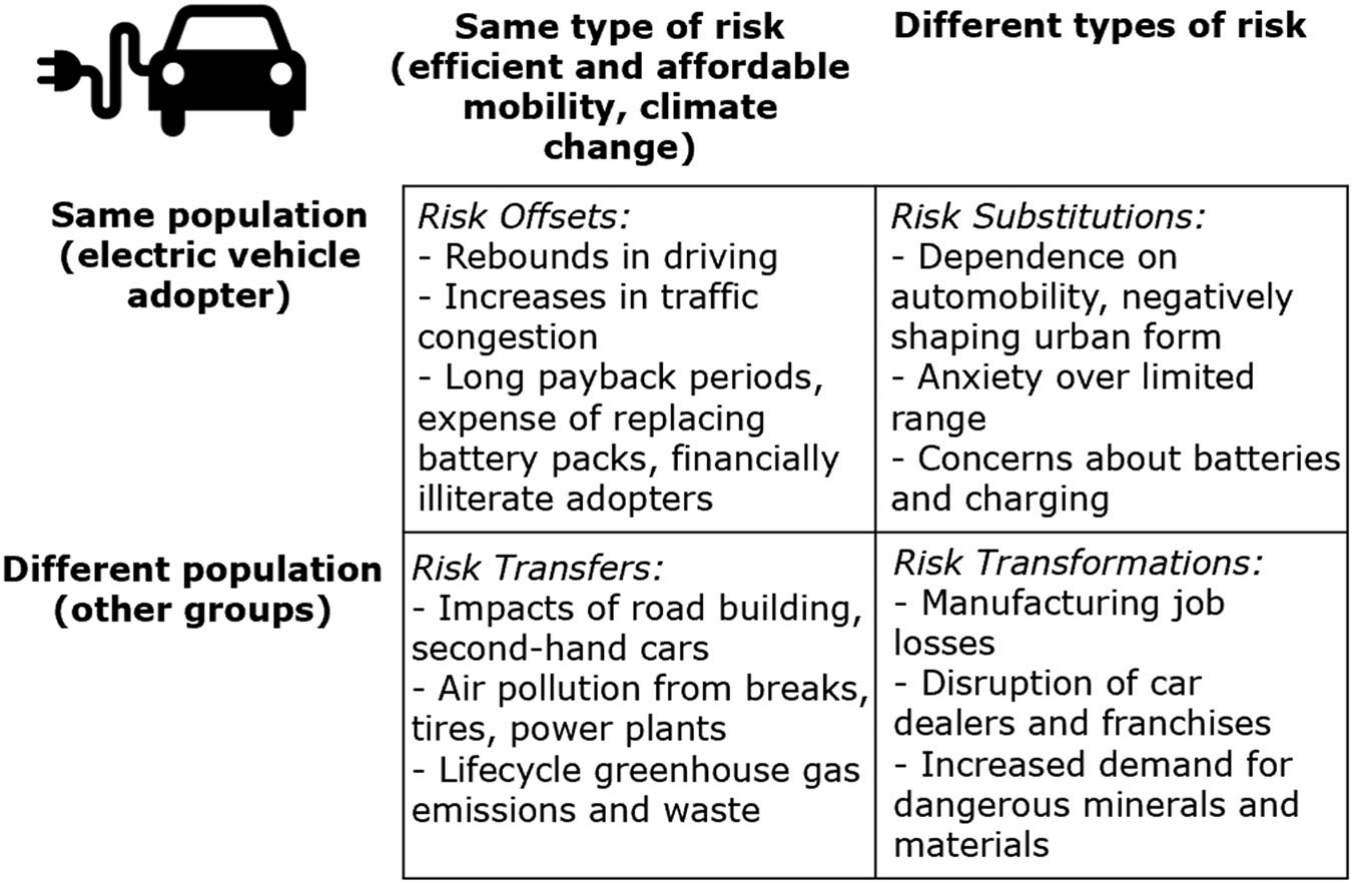
Depicting 12 risk–risk tradeoffs with battery electric vehicles (EVs). *Source*: Author.

Battery EVs are advocated for on behalf of their ability to lower carbon emissions and improve the reliability and efficiency of personal transportation. But evidence of Risk Offsets is apparent (see Figure 5). EVs run the risk of further embedding motorized, private automobility, which can contribute to congested roads and traffic jams and longer commuting times. Rebounds in driving times and distances are common, with Graham‐Rowe et al. (2012) noting that because adopters perceive their cars to be more “environmentally friendly,” they are driven almost 65% more than older conventional cars. Similar increases in distances traveled post‐EV adoption have been confirmed by multiple other studies (Langbroek et al., 2018; Seebauer, 2018; Sovacool et al, 2019a) and in some contexts can result in increased overall net carbon emissions for transport (Hamamoto, 2019). Being generally more capital‐expensive upfront than a conventional car, EVs also require longer payback periods, leading to more uncertain payback times and the risk that EVs become a financial burden if warranties expire or battery packs need to be replaced unexpectedly—a problem aggravated by financial illiteracy and a general trend for consumers not to calculate the benefits of fuel savings or economy appropriately (Geller & Attali, 2005; Greene et al., 2007; Turrentine & Kurani, 2007).

Risk Substitutions materialize. Because EVs embed dependence on automobility and passive transport, they tend to shape infrastructural and urban development patterns in favor of cars, and thus create cities that need roads and parking lots but generate spaces that are less amenable for walking, active transport, or public transport, which affects everyone (Kester et al., 2020). In many urban communities, EVs and roads are impinging on many of the spaces needed for other forms of green mobility, including cycle tracks, bus lanes, and walking paths (Henderson, 2020). EVs can contribute to psychological distress via “range anxiety,” concerns about how far they can be driven before being recharged or depleted (Egbue & Long, 2012; Franke & Krems, 2013; Franke et al., 2012; Tran et al., 2012) or frustration and concerns over battery degradation and the availability or affordability of charging infrastructure (Axsen et al., 2016; Gao & Kitirattragarn, 2008).

Risk Transfers are apparent, including the environmental impacts of road building to respond to greater EV demand. For instance, in Norway, planners are using EV adoption as an excuse to build new roads, even in restricted or sensitive areas such as wetlands (Sovacool et al., 2019b). The environmental impacts of those roads can include embodied emissions from concrete and asphalt as well as direct emissions from construction (Marzouk et al., 2017) in addition to the risk of landslides and flooding (McAdoo et al., 2018). The conventional cars that EVs displace do not immediately disappear and are instead often resold on secondary markets in countries with fewer environmental restrictions, such as those in South America or Sub‐Saharan Africa (Dall‐Orsoletta et al., 2022). Moreover, EVs do not eliminate air pollution. Although they do displace it from automotive tailpipes, it is shifted to power plants, which can then expose communities living near those facilities to higher levels of particulate matter pollution and sulfur dioxide emissions (Kintner‐Meyer et al., 2007). Moreover, recent studies show that the mass of particulate matter pollution from tires and brakes—which EVs still depend upon and contribute to—far exceeds the mass of emissions from tailpipes, in some cases being 100 times greater (Robbins, 2023). Additionally, the majority of EVs rely on high‐voltage lithium‐ion batteries that are difficult to recycle and that generate their own waste streams including carbon emissions near lithium mines, which will eventually require energy‐intensive car dismantling, scrapping, and recycling (Morse, 2021; Skeete et al., 2020).

Finally, Risk Transformations are perceptible. Widespread adoption of EVs correlates with manufacturing job losses for conventional automakers, especially involving the making of engines and traditional automotive parts. The Society of Motor Manufacturers and Traders has estimated that 15% of production jobs in the automotive sector are at risk of being lost, especially related to engines, fuel tanks, and exhaust systems (Campbell, 2022). EV adoption is perceived as harmful for car dealerships and franchises because EVs take longer to sell than conventional cars (customers have more questions and require repeated test drives) and have far fewer maintenance needs, even though most franchise revenues now come from conventional car maintenance (Cahill et al., 2014; Matthews et al., 2017; Zarazua de Rubens et al., 2018). Furthermore, the production and manufacturing of EVs is accelerating resource and energy demand, which intensifies reliance on unfair and exploitative mining practices for critical materials such as rubber, bauxite, lithium, or cobalt (Chason & Sharrock, 2023; Henderson, 2020; Sovacool, 2019).

### 4.3 Household energy efficiency retrofits

**FIGURE 6:**
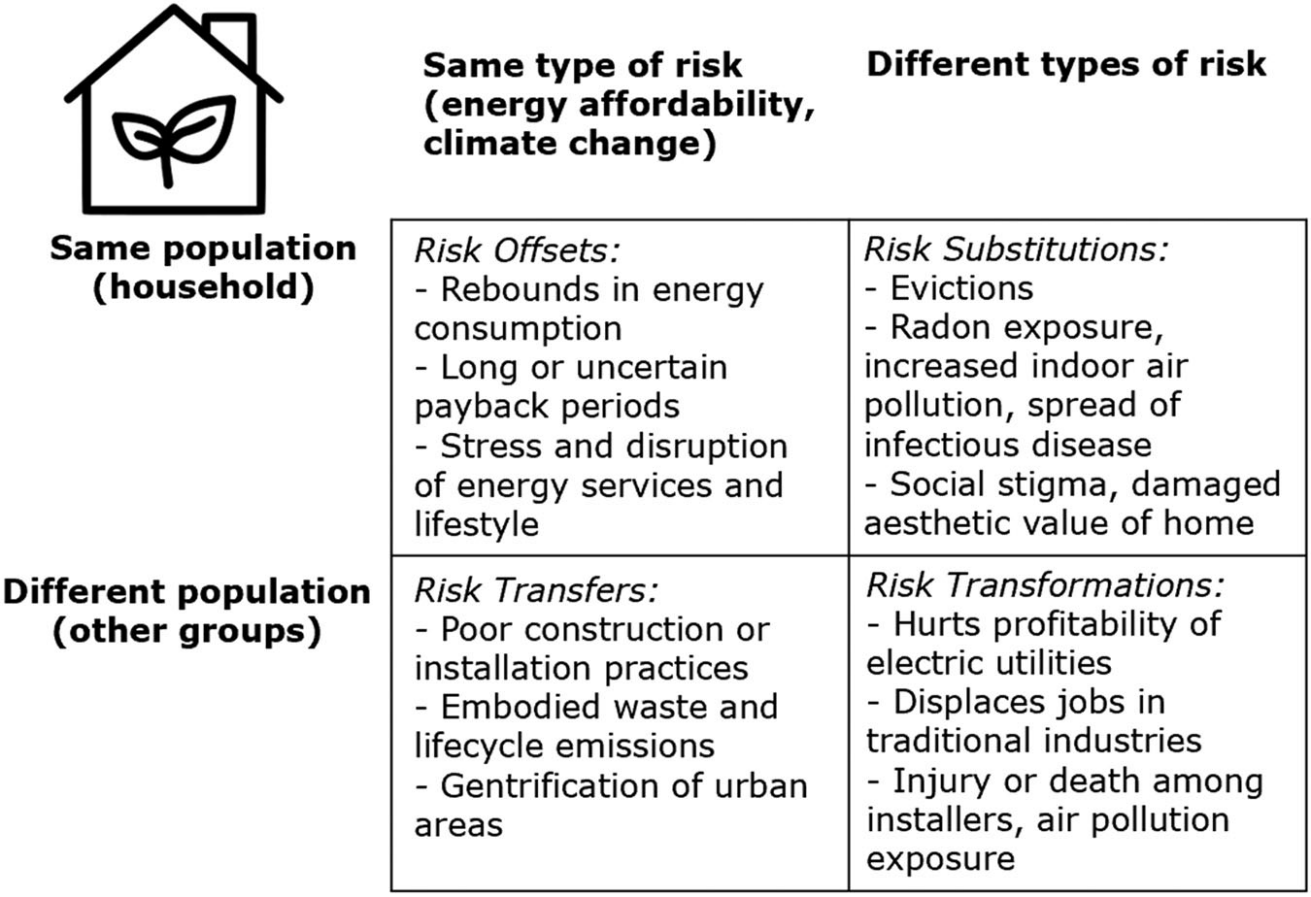
Depicting 12 risk–risk tradeoffs with household energy efficiency retrofits. *Source*: Author.

The twin target risks for retrofits are energy affordability and climate change. But Risk Offsets are discernable in terms of rebounds in energy consumption (see Figure 6). This rebound or “take‐back” effect manifests directly through amplified energy usage after a retrofit and indirectly through additional consumption that may come from future energy or monetary savings (Chitnis et al., 2013; Greening et al., 2000; Hong et al., 2006; Owen, 2010). Research in the United Kingdom has quantified these rebounds, finding that on average, total energy consumption per household can rise after a retrofit is completed (Green & Gilbertson, 2008). The economically or socially privileged have more efficient technologies or homes but still tend to use more total energy regardless (Chen et al., 2022; Goldstein et al., 2022). Long and uncertain payback periods also exist for efficiency investments, and in some situations, their presumed benefits are not worth the initial cost of investment (Togashi, 2019). The installation of cavity wall insulation, for example, can take 30–40 years to pay for itself, when many households expect payback periods of 3 years or less (Geller et al., 2005). Stress and disruption of energy services and lifestyle can occur, even to the point where these factors convince households to abandon a retrofit project after it has started (Collins & Curtis, 2017).

Risk Substitutions are noticeable and can be severe. Retrofits can contribute to housing precarity and “renovictions,” where owners or landlords evict poor occupants out of homes so that a renovation can be conducted, or after the fact, due to rent increases after a retrofit project is completed (Bartram, 2023; Kurmayer, 2022; Polanska & Richard, 2021). Retrofitted homes can introduce acute and adverse health effects including allergic reactions, elevated radon exposure, and the spread of infectious diseases. For instance, the Institute of Medicine (2011) warned that some retrofits caused mold‐causing dampness, poor ventilation, excessive temperatures, and emissions from building materials that may contribute to health problems. They can also affect indoor air quality and lead to excess radon exposure (Symonds et al., 2019) or trap smoke or unsafe dust particles from cooking in the home (Jürisoo et al., 2019). Some of the “energy‐saving” appliances installed with retrofits can rely on ecofriendly cycles with lower water temperatures that do not adequately kill dangerous pathogens, leading to exposure to potentially lethal germs and resulting infections (Schmithausen et al., 2019). Another Risk Substitution is that retrofitted energy‐efficient homes can carry a social stigma that targets homes that are “low class” due to their affiliation with low‐income weatherization and assistance programs or are seen as socially undesirable for degrading the aesthetic beauty or cultural heritage of a home (Sesana et al., 2019; Wilk & Wilhite, 1985).

Risk Transfers related to retrofits include poor construction or installation practices, which can devalue a home and create a risk for whomever purchases or occupies it after the retrofit is completed. Manuel (2011) documented problems of poor‐quality work in weatherization and retrofit programs and noted that some are caused by fly‐by‐night contractors (e.g., new or low‐quality installers) but that even high‐quality and experienced firms were not adequately training new workers. Inspections uncovered several instances of hazardous conditions created or worsened by retrofits, substandard work, and malfunctioning heating systems. Retrofits create a large amount of concrete, ceramic, metal, and timber waste, which are not always discarded properly or sustainably (Sáez et al., 2018). Li and Yang (2014) posit that retrofits are more difficult than erecting new buildings given that the impose greater space constraints on occupants and builders, and this makes the process riskier, more complex, less predictable, and more poorly planned—which all contribute to inadequate waste disposal practices, especially for internal fittings and finishes. The further gentrification of urban areas is a final Risk Transfer, as the need to improve the energy performance of residential buildings can have the macroeconomic effect of increasing property values and further segregating the housing market, displacing lower‐income, often non‐White, residents as a result (Bouzarovski et al., 2018; Grossmann, 2019; Rice et al., 2020).

**FIGURE 7:**
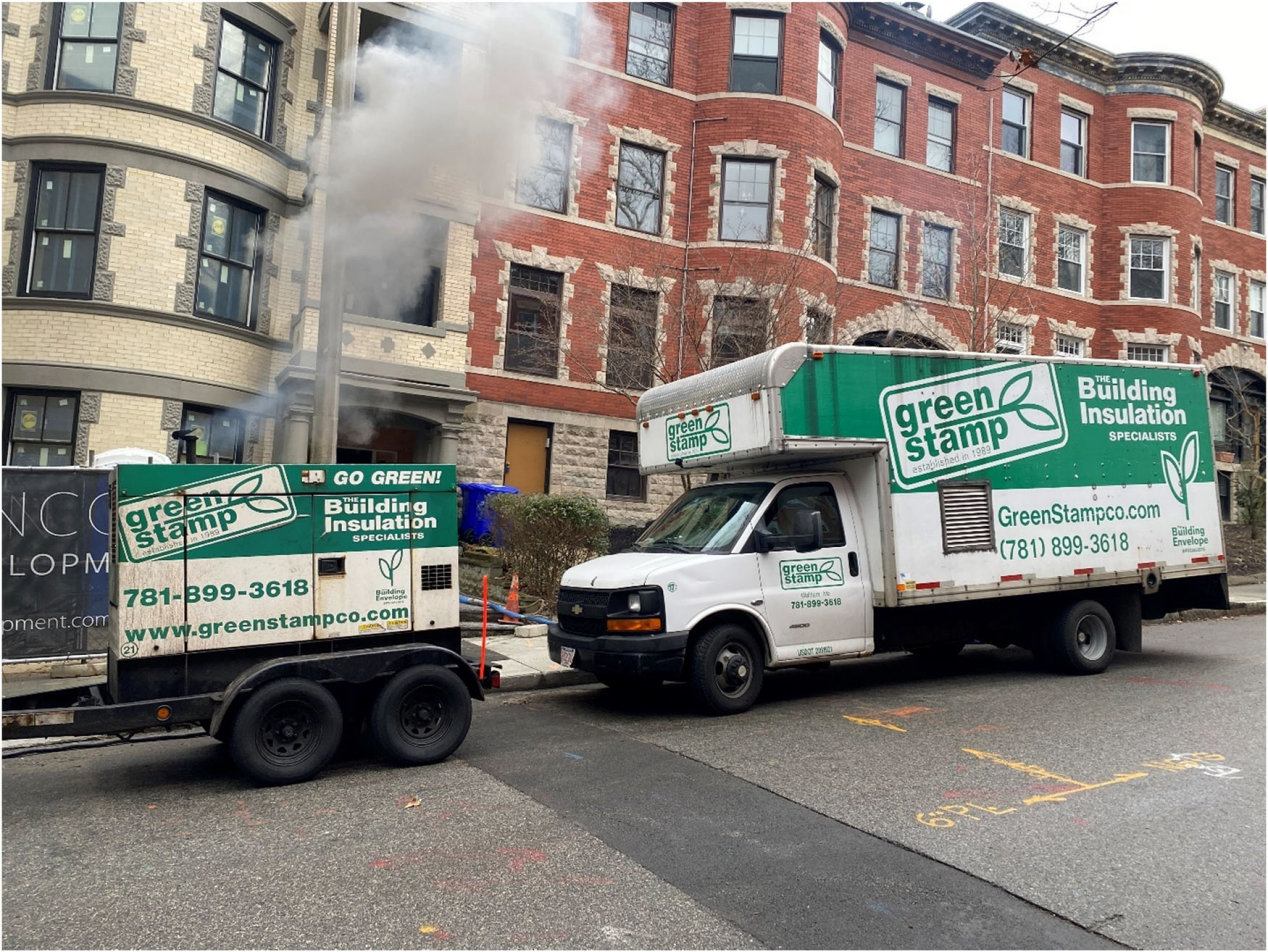
Air pollution exposures from building insulation retrofitting in Boston, MA, 2024. *Source*: Author.

Risk Transformations with retrofits include hurting the profitability of electric utilities that depend on maintaining or growing their electricity or natural gas sales. When rebound effects do not occur to a significant degree, retrofits make homes more efficient and contribute to a utility “death spiral” as customers consume fewer units of energy at their homes (Bischof, 2014; Cooper, 2017; Costello, 2014). Municipal gas utilities in the United States have opposed retrofit and efficiency efforts because they are perceived to “destroy markets” for gas by simultaneously lowering demand and price (Pipeline & Gas Journal, 2007, p. 29). Disruption is not limited only to electricity suppliers; energy efficiency efforts can also displace jobs in other traditional energy industries (Stern et al., 2016). Retrofitting can lastly be dangerous work for installers, with common accidents including falling from heights, falling objects, hazards of using old materials and power tools, and hitting/bumping the head and bruises (Nadhim et al., 2018). In serious cases, these result in death, with one study estimating 2.6 deaths per year in the Canadian retrofitting and building sector in Quebec (Gonzalez‐Cortes et al., 2021). Moreover, some retrofit operations in the United States still rely on portable diesel generators to provide energy for tasks such as foam insulation or cavity wall insulation—which generates their own localized forms of particulate matter pollution, creating an air pollution risk for the community (see Figure 7).

## 5 DISCUSSION: COMPLEXITIES, CHALLENGES, AND RISK RESOLUTION

In previous risk research (see especially Section 3.4), most risk transfers are treated as isolated to one quadrant of the Graham and Wiener (1995) typology. The examples in this paper show a mosaic of risk types, mediums, and transfers that crisscross this typology. In this section of the paper, the paper builds on these dynamics to discuss complexities and challenges, offers a framework for resolving tradeoffs, and sketches emergent research gaps.

### 5.1 Complexities and challenges

**FIGURE 8:**
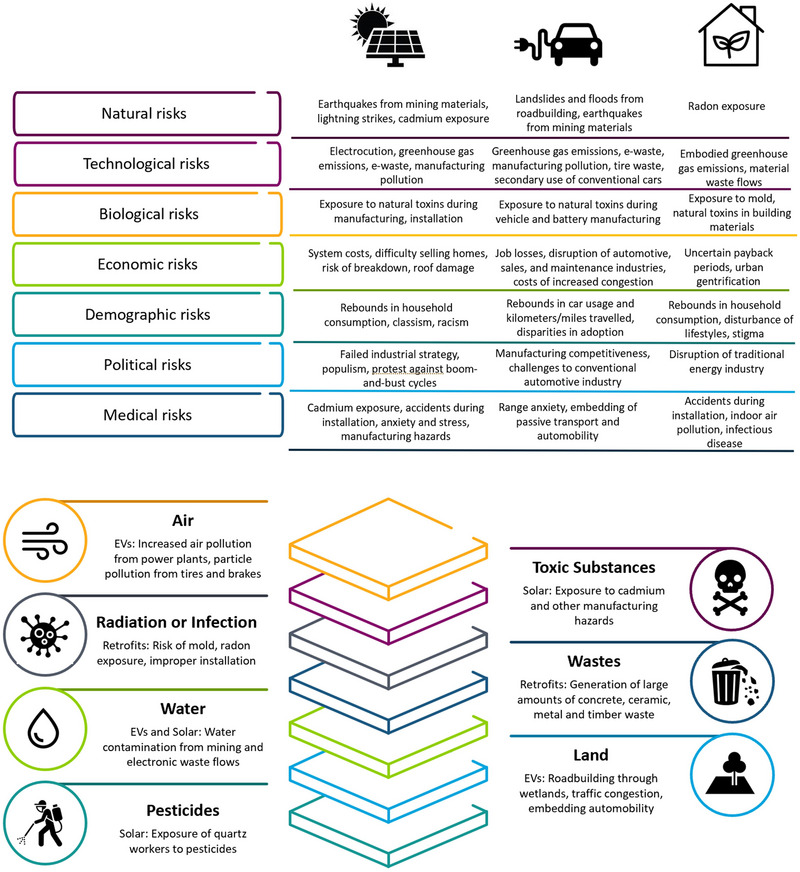
Categorical risks and risk mediums with solar energy, EVs, and retrofits. *Source*: Author. Specific examples given are not exhaustive, and more details are offered in Section 3.

As evidenced in Section 3, solar energy, EVs, and household retrofits all involve different categories of risk plus a diversity of risk mediums. As Figure 8 summarizes, these innovations can pose natural risks such as landslides or floods or exposure to radon. Technological risks include industrial pollution and waste, carbon or noxious emissions, electrocution, and manufacturing hazards. Biological risks include exposure to natural toxins or mold. Economic risks include capital and system costs, the risk of breakdown, damaging homes, job losses, market disruption, the costs of traffic congestion, and gentrification. Demographic risks include rebounds in household consumption, the perpetuation of racist or classist modes of adoption, or subjecting adopters to social stigma. Political risks include industrial strategy and competitiveness, populism, and protest. Medical risks include physical accidents and injury or occupational hazards, or mental health concerns including anxiety and stress, and in some situations, death.

Figure 7 also illustrates the different mediums that can deliver these categorically distinct risks. Some such as air pollution from power plants or tire pollution from EVs can be delivered via the air, and others such as exposure to mold or radon are spread via infection. Water can disseminate natural and synthetic toxins, whereas pesticides and toxic substances can affect miners and laborers at manufacturing facilities through exposure. Waste flows can involve concrete, ceramics, metal, and wood, which can be incinerated, end up in landfills, or contribute to electronic waste flows, and land can be a medium for embedding automobility, extracting precarious minerals and metals, or leading to more contested urban spaces and streets.

Such risks also have inherently distinct temporalities, with some occurring chronically (such as occupational accidents at factories), compared to some occurring catastrophically (accidents or lightning strikes among solar installers or retrofit operators). Some occur in the present or near term, such as an immediate increase in mining to create materials for solar panels, EVs, or energy‐efficient appliances, whereas others (such as waste streams) are shifted to future generations.

Using the typology of risk tradeoffs reveals the kinds of tradeoffs that can occur and begins to paint a picture of their severity, but it does not offer a framework to determine how they can be resolved. We turn to this final aspect in the next subsection.

### 5.2 A framework for resolving or weighing risk–risk tradeoffs

Faced with confronting the diversity of risk categories, mediums, severities, and tradeoffs with solar energy, EVs, and retrofits, decision‐makers must exercise reflection, care, and judgment when determining the optimal course of action. How should one handle a complex comparison of multidimensional risk portfolios? What factors should decision‐makers depend upon to guide their comparison of risk–risk tradeoffs? According to Graham and Wiener (1995), the decision‐maker should consider the attributes of target risks, countervailing risks, risk category, risk medium, and risk severity according to six criteria:

Magnitude or probability of risk: When both target and countervailing risks are compared, the magnitude and probability of those risks can differ greatly. As Graham and Wiener (1995) argue, the elevation in the probability of death from contaminants in fish is real and deserves consideration, but it is far smaller than the benefits gained by eating fish instead of meat. Although there is no universal threshold, or magic equation, that can hold true in every risk–risk case, and although judgments and uncertainties will always exist, efforts at estimating magnitude and probability must remain a central consideration for risk–risk assessment.

Size of population exposed: Even if the probability of a target risk or countervailing risk is held constant, the relative size of the exposed population is a compelling consideration. If 1000 homeowners completing a retrofit each face an incremental lifetime cancer risk of one in a million, the population risk (0.001 cases of cancer) seems negligible. If, however, 200 million citizens are exposed to that risk, the resulting population risk (200 cancers) is no longer trivial. An explicit recognition of the size of exposed populations can help anticipate and then avoid risk transfers.

Certainty in risk estimation: Scientific, lay, and even knowledge can all be imperfect, and consideration needs to be given to the quality and strength of evidence and the veracity of lived experiences, as well as the credibility of those sources. Some risk estimates may be hypothetical and speculative, others robustly supported and empirical. Where relative uncertainty or certainty can be quantified, such risk estimations should be adjusted accordingly. In making such comparisons, symmetrical reporting of uncertainty for both target and countervailing risks should be made; if worst‐case estimates are reported for one target risk, worst‐case estimates should be reported for countervailing risks as well.

Severity and subjectivity of adverse outcome: For many kinds of risk–risk tradeoffs, net‐risk calculations are exceedingly difficult because of different risk endpoints and variabilities in risk dissemination. Furthermore, some risks can fall into utilitarian cost–benefit calculations, but others cannot. Informed and more pluralistic value judgments must therefore be made to help weigh and measure dissimilar risk profiles.

In other words, risks differ not only by category and medium but are starkly different in terms of their consequences. Some, such as anxiety, stress, or frustration, are fundamentally different from species extinction, death, or serious debilitating injury. Some are systemic and chronic, such as the perpetual risk of inhaling mold in a retrofitted home, whereas others are catastrophic single events such as falling off a roof or being struck by lightning while installing a solar panel. Indeed, Table 2 indicates no less than 10 different attributes of risk impacts that can complicate any attempt of comparison or critical analysis. Table 2 offers a starting point for such a weighing of risk as it already begins to unpack severity according to the nature of risk, permanence, duration, temporality, incidence, equity, source, freedom, existing understanding, and relation to status quo as well as risks are visible (fires, floods) or invisible (microbes, radiation, fine particle pollution), among other attributes. Some sources of risk could even contribute to both at the same time, for example, an operational coal mine visibly causing traffic accidents and noise but invisibly causing arsenic pollution.

**TABLE 2:** Fifteen fundamental attributes of risk severity and consequence.

*Nature of risk*	Dreaded	Acceptable
*Permanence*	Irreversible and uncontrollable	Reversible and controllable
*Duration*	Chronic, systemic, and recurring	Catastrophic, probabilistic, and a single event
*Temporality*	Faced by future generations	Faced by those now living
*Incidence*	Retrospective, empirical, and historical	Prospective and hypothetical
*Equity*	Unfairly distributed	Fairly distributed
*Source*	Human‐made	Found in nature
*Freedom*	Voluntarily incurred	Forced exposure
*Existing understanding*	Known to science	Unknown
*Relation to status quo*	New	Old
*Observability*	Visible or explicit	Invisible or tacit
*Effect*	Immediate	Delayed
*Controllability*	Easily reduced	Not easily reduced
*Severity*	Fatal	Not fatal
*Intimacy*	Affects me or my loved ones	Does not affect me or my loved ones

*Source*: Modified from Sunstein (1996) and Fischhoff et al. (2013).

These fundamental and differentiating aspects of risk make conducting a risk analysis, or any sober comprehensive examination of risk–risk tradeoffs, exceedingly difficult and value‐laden (Esty, 1996; Rascoff & Revesz, 2002). Risk management is difficult to implement and enforce, as it can generate substantive uncertainty over not only *what* can be gained or lost by managing risk tradeoffs but *who* gains and loses, as well as *how* those decisions are made (Congressional Research Service, 2011). Any campaign for climate action that manages such a diffuse array of impacts risks waging a war with itself, clearing away target risks only by reaping a crop of countervailing risks of differing categories, mediums, and consequences.

**Distributional considerations**: Even when risk tradeoffs may involve equal probabilities and similarly severe adverse outcomes, who incurs the risk, when risks are unevenly distributed among the population, must be considered. Risks incurred by disadvantaged groups in society may deserve more consideration than risks incurred by all groups in society. Furthermore, one must seek to avoid the problem of “omitted voices” where some affected parties are absent from the decision process and have risks unduly imposed on them.

**The timing of risk impacts**: The timing of target and countervailing risks may differ and deserve consideration as well. The risks of injury or trauma may be more immediate and imminent than say the risk of cancer, which occurs only after a much longer latent period. Risks to future generations may also be distinguished from risks to the present generation.

**FIGURE 9:**
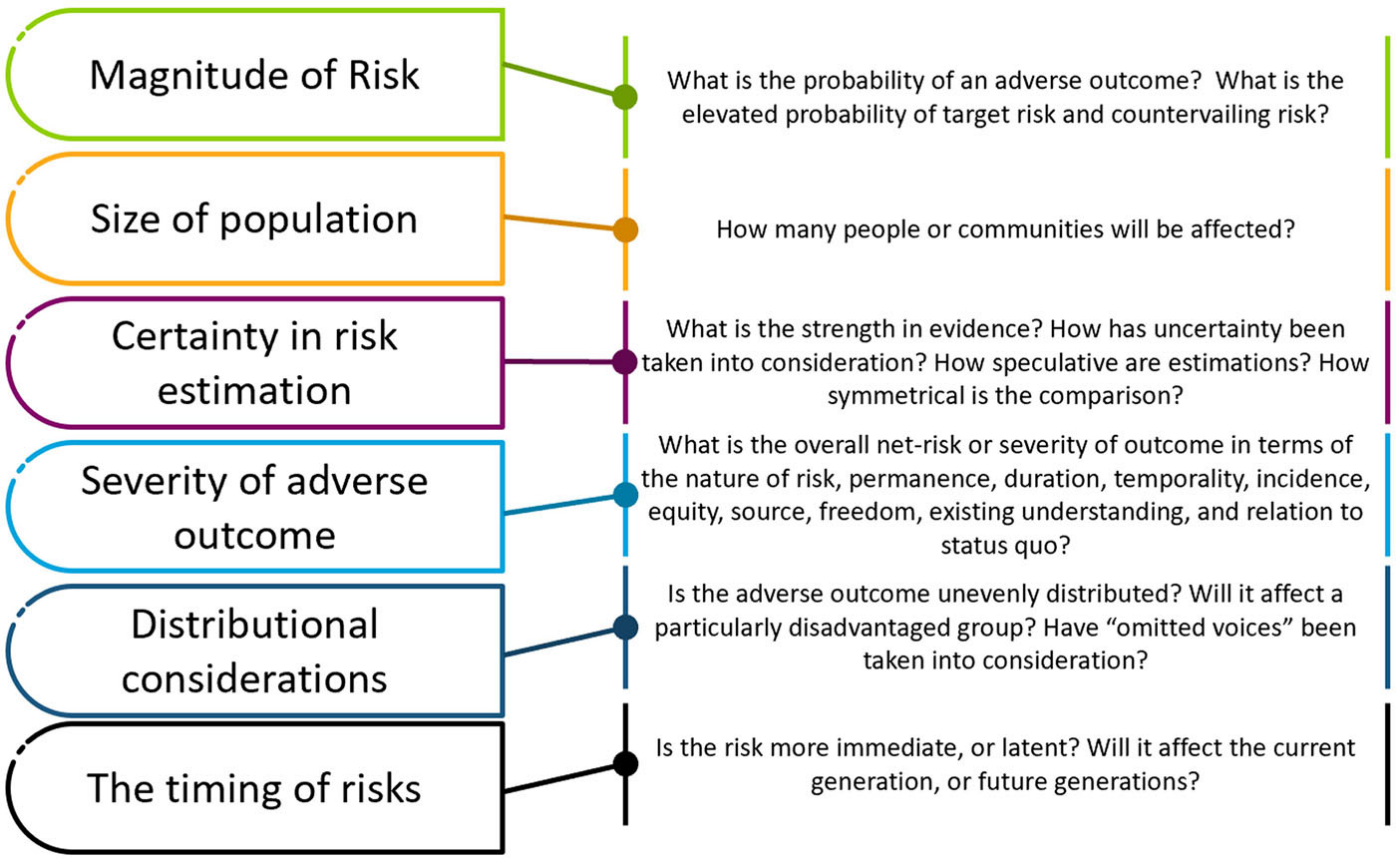
A framework to guide decision‐makers grappling with risk–risk tradeoffs. *Source*: Adapted from Graham and Wiener (1995).

These six criteria lead to a framework shown in Figure 9, which suggests that decision‐makers ask 13 specific questions.

### 5.3 Future research gaps

As complex as the risk–risk tradeoffs associated with solar energy, EVs, and retrofits are (see Section 5.1), and as much as the framework in Section 5.2 aspires to accomplish reflexive thinking, multiple research gaps exist. This study has mapped risk–risk tradeoffs for each low‐carbon in isolation from each other, but it is equally plausible that some households, and especially some communities, may see all three options being adopted or implemented together. That is, some households may co‐adopt solar panels, purchase an EV, and undertake a retrofit simultaneously. Moreover, this study has mapped only the risk–risk tradeoffs from the adoption of these three interventions but not the risk–risk tradeoffs from continued, aggravated climate change that could exist from *not* adopting them. Here, the net social gain or reduction in societal risk from adoption may outweigh the risk–risk tradeoffs shown here; there are many contexts and examples of where solar energy, EVs, and efficiency are *good* things for society. Last, there are dozens of other climate interventions, from hydrogen fuel cells to carbon capture and storage systems to wind farms and nuclear power plants, that also are deserving of risk–risk analysis alongside a comparison to conventional sources of energy such as oil wells, coal‐fired power plants, and natural gas processing stations. All these limitations point toward the need for more sophisticated, integrated, multi‐scalar, and multi‐temporal applications of risk–risk assessment.

A second research agenda concerns questions around better ways of assisting decision‐makers with how to identify, grapple with, and eventually resolve risk–risk tradeoffs. Most basically, and tragically: can the policymaking community ever move from the redistribution of risk transfers and adverse outcomes to the elimination of risk transfers and adverse outcomes? Is it possible to move toward some kind of dashboard, or decision‐making system, to allow a local, state, or national policymaker to evaluate all tradeoffs, mediums, and transfers involved, perhaps through a risk matrix? Might city, state, or federal planners and regulators benefit from risk register training, and if so, what would make it effective? Similarly, what might “risk‐aware” policymaking look like, and are there examples of where it has reduced the severity or quantity of risk–risk tradeoffs? Could future research efforts bring in notions from finance and asset pricing, where what matters is covariation and valuation of payoff of assets, so that net risk is better understood at a societal level? How might policymaking efforts be better informed by other research designs (different than those of document analysis utilized in this study) such as expert interviews, game theory, simulations, exercises, or deliberative discussions? Or last, given that risks are not fixed but instead relational and dynamic, how can the policymaking community use improved information to better hedge and eliminate risks—could self‐governing arrangements exist that minimize the persistent presence of risk, even in the absence of policy or in areas prone to weaker forms of governance?

## 6 CONCLUSION

**FIGURE 10:**
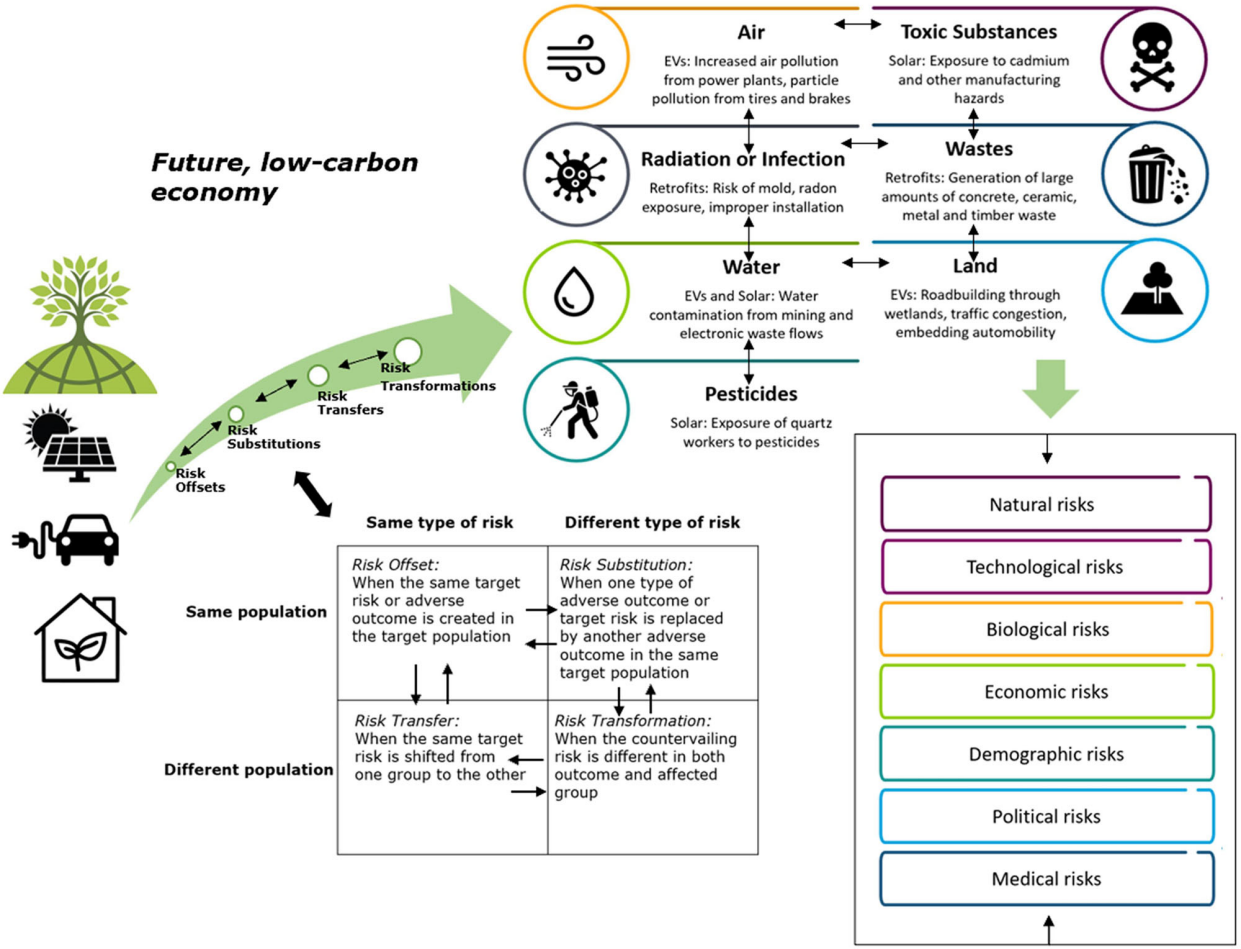
The risk–risk tradeoffs, mediums, and categorical consequences of solar energy, EVs, and retrofits. *Note*: The bidirectional arrows indicate risk transmission across types of transfer, mediums, and risk categories, resulting in a three‐dimensional matrix of risk shifting. *Source*: Author.

A future low‐carbon world will invariably depend on more distributed solar energy, the electrification of mobility, and more efficient homes and buildings. However, as solar panels, EVs, and retrofits are adopted, they create a low‐carbon risk society, as they enlarge a nest of adverse outcomes that transcend risk tradeoffs, mediums, and categories (see Figure 10). They can transfer and substitute target risks for target populations, for example, low‐carbon adopters and affluent households, but also transfer and transform countervailing risks for other populations, such as workers or communities of color. They can involve a multiplicity of mediums of distribution including air, radiation or infection, water, pesticides, toxic substances, wastes, and land, at times morphing risks from air to water (air pollution control equipment at power stations charging EVs that generate toxic sludge), pollutant to pollutant (carbon dioxide to emissions of methane and manufacturing gases), or from land to air (EVs discharging particles from their tires and brakes). They can consecrate a cornucopia of risk categories including landslides and floods, industrial pollution, the spread of natural toxins, lost jobs, dependence, eroded national competitiveness, and degraded health. The arrows in Figure 10 illustrate at least three elements of risk–risk conveyance: between transmission types (offset, substitution, transfer, and transformation), mediums such as air water and land, and categories such as biological, medical, or technological. Moreover, the presence of these risks associated with three low‐carbon innovations may explain why climate mitigation has seen its progress so frequently stymied, as climate action can be criticized by opponents for always presenting some form of new risk, or adversely affect some new group. Risk management therefore becomes a three‐dimensional dilemma entailing not only risk transmission but medium‐shifting and redistributing risk across different categorical types.

In the face of these immense and complex risk–risk tradeoffs, the response should not be nihilism or inaction but a more refined risk assessment that better accounts for decision‐making considerations such as the magnitude or probability of risk, size of population exposed, certainty in risk estimation, severity of adverse outcome, distributional considerations, and the timing of risk impacts, an approach unveiled in Section 5.2. The analysis undertaken here is furthermore exploratory, and as Section 5.3 on future research gaps suggests, other scholars are actively encouraged to build on and extend this work to other dimensions of risk, to other low‐carbon innovations, or to other research designs and calls to integrate risk assessment with decision‐making science.

Effectual climate risk governance and minimization become akin to three‐dimensional chess. Perhaps it is even like playing three‐dimensional chess on a constantly rotating board, on a moving train, in a dark tunnel with the lights out. Reflexive risk management in a low‐carbon future is both about eliminating risk but also how best to manage a collection of compelling yet capricious risk–risk tradeoffs. Risk–risk decision‐making needs to become more intelligent, more reflective, and more effective. Both the scope and severity of target and countervailing risks, and our interventions to address them, are entirely within our power and agency to change.

## APPENDIX 1

### 1.1 Seventy‐five examples of risk–risk tradeoffs (in alphabetical order by example)

**Table:**

No.	Example	Description	Target risk	Countervailing risk	Category	Medium of transfer	Reference(s)
1	1977 Clean Air Act	Required that all coal burning power plants installed scrubbers to reduce the risk of sulfur dioxide, a criteria air pollutant, but generated tons of toxic sulfur sludge that had to be disposed of elsewhere	Air pollution	Water and land pollution	Technological	Water	Graham and Wiener (1995)
2	Acid rain abatement	Abatement of nitrogen oxide can reduce the risk of acid deposition but increase the risk of concentrated deposits in soils and watersheds; due to successful reductions in direct anthropogenic emissions of nitrogen oxide, the role of background sources from soils (both natural and agricultural) is increasingly important to the atmospheric chemistry that leads to worse ozone pollution	Air pollution	Land and water pollution	Technological	Water	Campbell et al. (2019); Geddes et al. (2022)
3	Airbags	Can guard against death and severe injury from high‐speed collisions but can also kill the elderly and fragile children	Automobile safety	Automobile safety	Technological	Technology	Hansen et al. (2008)
4	Antidepressant drug treatment	The use of anti‐depressant drugs such as Prozac and Elavil can lower the risk of depression but increase the incidence of cancerous tumors	Depression	Cancer	Medical	Toxic substances	Nemecek (1994)
5	Artificial sweeteners	Bans on cyclamates and saccharin can reduce the risk of cancer but lead to increased consumption of sugar, with attendant risks of weight gain and diabetes	Cancer	Weight gain, diabetes	Medical	Toxic substances	Graham and Wiener (1995); Hansen et al. (2008)
6	Automated vehicle adoption	Self‐driving cars can reduce the incidence and severity of car crashes but also lead to situations of possible sexual harassment and rape	Automobile safety	Human health	Technological	Technology	Blyth (2019)
7	Avoiding eating fish	Avoiding fish can reduce the intake of methylmercury and other pollutants to pregnant women and unborn children but increase the risk of heart disease and omega 3, which help improve the central nervous system of babies	Maternal and prenatal health (reduced exposure to dioxins, mercury, and pollutants)	Maternal and prenatal health (increased risk of cardiovascular disease)	Medical	Toxic substances	Hansen et al. (2008)
8	Ban of antibiotic growth promoters in animal feed	Intended to reduce the development of resistance bacteria in livestock, it has adverse effects for human and animal welfare	Animal and human safety	Animal and human safety	Biological	Pesticides	Hansen et al. (2008)
9	Ban on chlorofluorocarbons (CFCs)	Required the substitution of ozone‐depleting substances, which aggravated the use of F‐gases and refrigerants with high global warming potential	Ozone depletion	Climate change	Technological	Radiation or infection	Graham and Wiener (1995)
10	Banning ethylene dibromide	Its ban as a fungicide reduced the risk of cancer but increased the risk of fungus on grains and nuts that generated a more carcinogenic aflatoxin	Illness	Illness	Biological	Toxic substances	Graham and Wiener (1995); Hansen et al. (2008)
11	Banning leaded paint in buildings	Removes lead from households but increases occupational exposures	Lead poisoning among homes	Lead poisoning among workers	Medical	Toxic substances	Graham and Wiener (1995)
12	Banning nitrates in food	Nitrates in food are known to react with other food agents to form nitrosamines, which are carcinogens, but banning them increases the risk of deadly botulism in canned goods stored in airtight conditions	Cancer	Botulism	Biological	Toxic substances	Hansen et al. (2008)
13	Banning organophosphates (OP)/carbamate pesticides	Banning such pesticides can reduce acute neurotoxic effects to workers and longer‐term exposure to consumers but can increase the risks of outbreaks of pests and increase the costs of fruits and vegetables	Exposure to workers and consumers	Increases in pests	Biological	Pesticides	Hansen et al. (2008)
14	Banning the pesticide Alar (daminozide)	Was banned over concerns it exposed workers to environmental and health risks but increased labor and waste costs for apples, leading to decreased consumption, which also had negative health affects among consumers	Worker safety	Consumer health	Economic	Pesticides	Hansen et al. (2008)
15	Bans of chlorinated solvents in the vehicle repair industry	Reduced the environmental exposure of chlorine, but its alternatives increased risks of peripheral neuropathy in automobile technicians	Environmental pollution	Worker exposure	Technological	Toxic substances	Hansen et al. (2008)
16	Bans on asbestos in automobiles	Minimizes the risk of cancers in mechanics doing brake services but increases highway fatalities due to less effective brake linings, as asbestos brakes reduce the stopping distance of vehicles	Cancer	Automobile accidents	Technological	Toxic substances	Hansen et al. (2008)
17	Bans on asbestos in buildings	Its inhalation from housing insulation can increase the risk of cancer but removal also increases the risk of exposure to workers	Cancer	Exposure to workers	Technological	Toxic substances	Graham and Wiener (1995); Hansen et al. (2008)
18	Bicycle lanes	Bicycle lanes can motivate and increase the uptake of cycling but can lead to narrower and more congested roads, contributing to traffic jams and associated pollution	Active mobility, human health for cyclists	Increased traffic congestion and pollution among drivers	Technological	Air	Hancocks (2020)
19	Bioenergy with carbon capture and storage	Can enhance the ability for forests and ecosystems to biologically store and sequester carbon but at the risk of interfering with ecosystem services and promoting monoculture plantations	Climate change	Forest conservation	Technological	Land	Sovacool et al. (2023b)
20	Breastfeeding babies	Breastfeeding can help protect babies against some short‐ and long‐term illnesses and diseases but can also increase exposure to chemicals and toxins in breast milk	Newborn health	Newborn health	Medical	Toxic substances	Hansen et al. (2008)
21	Chemotherapy	Can minimize the risks of cancer but only via the risks of infection, hair loss, bleeding, anemia, and even death	Cancer	Illness	Medical	Toxic substances	Crawford et al. (2004); Lahart et al. (2015)
22	Child safety caps on medicine bottles	Decreased the risk of children opening a bottle of aspirin, but increased the risk of poisoning antibiotics from unregulated cleaning and polishing agents and complacency among parents (who would leave caps off or medicine cabinets unlocked)	Child safety	Child safety	Medical	Toxic substances	Graham and Wiener (1995)
23	Chlorinated drinking water	Policies to reduce the risk of cancer associated with chlorine can increase more immediate risks from water‐borne microbial diseases (such as cholera) that chlorine can kill	Cancer	Water‐borne diseases	Medical	Toxic substances	Graham and Wiener (1995); Harvard Center for Risk Analysis (1995)
24	Clean coal technologies	Can capture carbon dioxide from coal before or after combustion to reduce the risks of climate change but consumes more coal per unit of energy delivered (worsening mining risks) and consumes more water	Climate change	Coalmining and water use	Technological	Water	Sovacool et al. (2011)
25	Clozapine therapy	Can treat mental illness but only at the risk of physical health and injury	Suicide, schizophrenia	Agranulocytosis, seizure, infection	Medical	Toxic substances	Graham and Wiener (1995)
26	Coral reef protection in Australia	Efforts to use fogging and spraying, and modifying the genome of coral, can enhance the resilience of the Great Barrier Reef but release modified organisms into the environment and interfere with cloud forests	Environmental conservation, resilience to climate change	Release of toxins, impacts on cloud cover	Biological	Water, air	Sovacool et al. (2023a)
27	Delaney Clause	Seeks to reduce the risk of cancer from synthetic chemicals used in foods but not the cancer risk of natural chemicals found in foods, which can be even more harmful	Cancer	Cancer	Medical	Toxic substances	Ames et al. (1990a, 1990b)
28	Diapers	Disposable diapers increase sanitation but contribute to solid waste and landfills, but cloth diapers contribute to air pollution risks by requiring energy‐intensive washing and visits by washing services	Sanitation	Waste, energy consumption, water use	Technological	Water, air	Graham and Wiener (1995)
29	Domestic bans of dichlorodiphenyltrichloroethane (DDT)	Banning DDT over the risks of cancer only shifted the risk to increased use of OP/carbamate pesticides that also have cancer‐causing effects	Exposure to workers and consumers	Exposure to workers and consumers	Medical	Pesticides	Hansen et al. (2008)
30	Enhanced weathering	Can accelerate the ability for minerals, rocks, and fields to durably store carbon but at the risk of expanded mining operations and interference with the water supply	Climate change	Water availability and quality	Technological	Land, water	Sovacool et al. (2023b)
31	Estrogen therapy	Can reduce risks of heart disease and bone deterioration but at the heightened risk of cancer	Heart disease, osteoporosis	Uterine and breast cancer	Medical	Toxic substances	Graham and Wiener (1995)
32	Fuel economy standards for vehicles	Fuel economy standards led to more efficient automobiles with less tailpipe pollution and less dependence on overseas oil, but it increased the risk that vehicle manufacturers made smaller, lighter, less safe vehicles in crashes and that consumers kept older, less efficient cars contributing to air pollution longer	Fuel inefficiency, waste, oil dependence	Fuel inefficiency (for older cars), automobile safety	Economic	Air	Hansen et al. (2008); Rascoff and Revesz (2002)
33	Fuel‐substitution of coal with natural gas	Can lower the risk of carbon dioxide emissions but increase the risk of methane emissions	Climate change	Climate change	Technological	Air	Howarth (2014); Alvarez et al. (2018)
34	Genetically modified food in Zambia	Zambia rejected food aid from the United States over concerns that genetically modified corn would be harmful to its population but then increased food shortages and famine	Famine, food insecurity	Famine, food insecurity	Political	Land	Hansen et al. (2008)
35	Global bans of DDT	Bans reduced the risk of adverse environmental and health effects but increased malaria outbreaks killing millions	Exposure to workers and consumers	Increased outbreaks of malaria	Medical	Pesticides	Hansen et al. (2008)
36	Hazardous waste cleanup	Can reduce the risks of soil and groundwater contamination of chemicals to future generations but can also lead to an increased risk of accidental fatalities among cleanup crews	Soil and groundwater contamination	Occupational accidents and fatalities	Technological	Land, water	Graham and Wiener (1995); Harvard Center for Risk Analysis (1995); Hansen et al. (2008)
37	Health and climate impacts of carbon capture and direct air capture	Use of negative emissions technologies to curb climate change risks can lead to increased air pollution	Climate change	Air pollution	Technological	Air	Jacobson (2019)
38	Heath risks of hospitalization	Hospital care can reduce risks from trauma and illnesses but can also lead to other illnesses, injury, and iatrogenesis	Illness	Illness	Medical	Radiation or infection	Graham and Wiener (1995)
39	Hormone replacement therapy	Can decrease and prevent the risks of menopause (hot flashes, changes in vaginal walls, and the risk of hip fractures and osteoporosis) but increases the risk of cancer, cardiovascular diseases, stroke, and pulmonary embolus	Female health	Female health	Medical	Radiation or infection	Hansen et al. (2008)
40	Lead battery recycling	Recycling lead batteries removes them from landfills where they can expose communities to neurological and environmental risks but increases exposure to workers and residents who live near smelting facilities	Lead exposure to consumers and communities	Lead exposure to workers and communities	Technological	Toxic substances	Hansen et al. (2008)
41	Legalizing drugs, alcohol, or prostitution	Reduces the risk of crime but increases the risk of addiction and its health effects	Violent crime	Addiction	Demographic	Radiation or infection	Graham and Wiener (1995)
42	Light rail mass transit	Urban light rail mass transit systems like the London Underground can promote efficient use of public transport that is more environmentally friendly than cars but can also expose commuters to high levels of underground dust including carcinogenic particulate matter pollution	Traffic congestion, climate change	Human health	Technological	Air	Mayor of London (2022); Noble (2023)
43	Mandatory minimum legal sentencing	Can reduce the risk of keeping non‐violent criminals off the street, overcrowds prisons, and utilizes space that could be used to incapacitate more violent criminals	Non‐violent crime	Prison overcrowding	Demographic	Technology	Graham and Wiener (1995)
44	Mass inoculation against parasitic worms	Mass inoculation with antimony compounds for the treatment of endemic schistosomiasis (parasitic worms) can also lead to a higher incidence of Hepatitis C	Human health	Human health	Medical	Radiation or infection	Hansen et al. (2008)
45	Mobile phones	Can be used to more rapidly call emergency services in case of an accident, but also can contribute to crashes when used improperly while driving	Automobile safety	Automobile safety	Technological	Technology	CDC (2023)
46	Monuments in Washington DC	Repeated scrubbing and cleaning of the Jefferson and Lincoln Memorials has caused the monuments to crumble more aggressively than did the dirt and graffiti on them	Aesthetics	Architectural integrity	Natural	Toxic substances	Graham and Wiener (1995)
47	Ocean dumping of industrial wastes	Ban on ocean dumping can encourage disposal and incineration of waste on land, increasing health risks closer to populations and fragile freshwater ecosystems	Preservation of the ocean	Pollution on land and in freshwater ecosystems	Technological	Land	Schneider (1993)
48	Ownership of firearms	Gun ownership at home can defend against the risk of criminal intruders but increase the risk of accident gunshots within the family	Crime	Household safety	Demographic	Technology	Graham and Wiener (1995)
49	Prohibitions of drugs, alcohol, and prostitution	Reduces the risk of people suffering addiction but increases the risk of violent crime and bootlegging as competition for underground markets is waged, sometimes violently	Addiction	Violent crime	Demographic	Technology	Graham and Wiener (1995)
50	Public participation against pipelines	People engaging in public participation processes to reduce the risk of pipeline spills come to believe they have little or no effect on agency decision‐making, leading to significant harm to the mental and physical health of those individuals	Pipelines	Mental health	Political	Radiation or infection	Bell et al. (2024)
51	Public participation against shale gas in the United Kingdom	People engaging in public participation processes to enhance local democracy and stop shale gas extraction risks suffer anxiety, community alienation, and financial stress	Shale gas extraction	Mental health and household finances	Political	Radiation or infection	Sovacool et al. (2020)
52	Radiation therapy for prostate cancer	Men treated with prostate cancer with external beam radiation had an increased relative risk of rectal cancer	Prostate cancer	Rectal cancer	Medical	Radiation or infection	Hansen et al. (2008)
53	Recycling plastic water bottles	Recycling plastic bottles can address the pollution and waste crisis but can be more toxic to consumers due to higher levels of toxics, pesticides, and pharmaceuticals in recycled products	Environmental pollution, waste, circular economy	Human health	Technological	Pesticides	Carmona et al. (2023); Carney Almroth et al. (2023)
54	Reducing ground‐level ozone	Reducing ground‐level ozone can lower the risk of respiratory illness and premature mortality but increase exposure to ultraviolet radiation that causes skin cancer and cataracts	Respiratory illness	Skin cancer	Medical	Radiation or infection	Hansen et al. (2008)
55	Reformulated gasoline	Gasoline blended to burn more cleanly reduces the risk of smog‐forming and toxic pollutants in airsheds but increases health concerns such as nausea and cancer	Air pollution	Public health	Technological	Air	Harvard Center for Risk Analysis (1995)
56	Remediation of the ExxonMobil Valdez oil spill	The spraying of hot water to clean off Alaskan beaches in Price William Sound removed oil from the surface, reducing risks to otters and birds but killed marine organisms, harming the ecosystem's ability to recover	Oil cleanup	Ecosystem collapse	Biological	Water, land	Graham and Wiener (1995)
57	Replacing phosphorus in detergents with nitrilotriacetic acid (NTA)	Reduced the amount of phosphorus released into waterways contributing to eutrophication but increased exposure to NTA, which could be a human carcinogen	Water pollution, eutrophication	Cancer	Technological	Water	Hansen et al. (2008)
58	Restrictions on elderly drivers	Lessens the number of elderly drivers improving automobile safety but results in social isolation, depression, and declining health of the elderly, including injury, crashes, and falls when using alternative modes of transport such as walking or cycling	Automobile safety and crashes	Depression, social isolation, nutrition	Demographic	Radiation or infection	Graham and Wiener (1995); Hansen et al. (2008)
59	Ridesharing and carpooling	Ridesharing and carpooling can minimize drunk driving and the need to own a car (and contribute to its environmental impact) but can lead to increased consumption of alcohol and public intoxication (by increasing the ease, frequency, and intensity of drinking alcohol)	Automobile safety, efficiency, climate change	Human health	Technological	Radiation or infection	Axsen and Sovacool (2019); Teltser et al. (2021)
60	Seat belts	Feelings of safety and security engendered by seat belts lead to more dangerous driving	Automobile safety	Automobile safety	Technological	Technology	Evans (1985)
61	Stratospheric aerosol injection	Particles can be sprayed to reduce local or regional temperatures, offsetting heatwaves but can also be perceived to modify weather or create a weapon of war	Climate change	Weather modification, weaponization, and conflict	Technological	Air	Sovacool et al. (2023b)
62	Substituting coal‐fired electricity with nuclear power	Deploying nuclear power facilities to replace coal‐fired facilities can minimize the risks of coal mining and carbon emissions but increase the risk of nuclear accidents and waste	Coal mining accidents and black lung disease, climate change	Risk of nuclear accidents and accumulation of long‐lived nuclear waste	Technological	Radiation or infection	Sovacool et al. (2021); Wheatley et al. (2017)
63	Substituting leaded gasoline with methyl tert‐butyl ether (MTBE)	The use of MTBE as a replacement for leaded gasoline reduced lead exposure but increased chemical pollution of groundwater in urban areas of the United States	Airborne concentrations of lead	Groundwater contamination	Technological	Water	Hansen et al. (2008)
64	Substituting nuclear power with coal‐fired electricity	Deploying coal‐fired power stations to replace nuclear (e.g., after an accident) can lower the risk of future accidents but increase the risks inherent in coal mining and combustion	Risk of nuclear accidents and accumulation of long‐lived nuclear waste	Coal mining accidents and black lung disease, climate change	Technological	Land, Radiation or infection	Sovacool et al. (2021)
65	Substitution of ammonia, methyl chloride, and sulfur dioxide with CFCs	Reduced the risk of fires, corrosion, and contamination and spoilage of food but depleted the ozone layer, which protected humans against dangerous radiation	Fire risk, food contamination, refrigerant leaks	Ozone depletion	Technological	Radiation or infection	Hansen et al. (2008)
66	Sustainable forest management	Restrictions on the logging of American forests to safeguard wildlife and old‐growth habitats can increase timber prices and increase the risk of logging of rainforests overseas	Domestic logging	Overseas deforestation	Economic	Land	Graham and Wiener (1995); Lofstedt and Schlag (2017)
67	Synthetic chemical pesticides	Restricting their use can reduce the risk of toxin exposure to farmers but lead to shifts to plant‐based (non‐synthetic) pesticides that are even more harmful	Exposure to chemical toxins	Exposure to natural toxins	Biological	Pesticides	Graham and Wiener (1995)
68	Tamoxifen	Can reduce the recurrence of breast cancer but can increase the risk of uterine cancer	Breast cancer	Uterine cancer	Medical	Toxic substances	Graham and Wiener (1995)
69	Tube well drilling in South Asia	Millions of tube wells were drilled in Bangladesh, India, and Nepal to improve water quality and reduce the risk of diarrheal infection caused by sewage but only increased the risk of arsenic poisoning and cancer	Water supply and security	Arsenic poisoning	Technological	Water, toxic substances	Hansen et al. (2008)
70	Ultraviolet sunscreen	Prohibiting sun lotions that contain ultraviolet components can reduce estrogenic cancer risks but increase the risk of skin cancer when exposed to the sun without proper protection	Skin cancer	Skin cancer	Medical	Radiation or infection	Hansen et al. (2008)
71	Use of bromopropane in cleaning solvents	Provided an alternative to CFCs but increased the prevalence of ovarian failure in female workers	Ozone depletion	Worker exposure	Technological	Toxic substances	Hansen et al. (2008)
72	Use of hydrochlorofluorocarbon (HCFC) as an alternative to CFCs	Reduced the adverse effects of CFCs on the ozone player but led to increased risk of liver disease among workers exposed to HCFCs	Ozone depletion	Worker exposure	Technological	Toxic substances	Hansen et al. (2008)
73	Vegetarian and pescatarian diets	More plant and fish‐based diets are good for the heart but can create risks of exposure to other pollutants, especially for pregnant women	Heart disease	Cancer, pesticide poisoning, mercury exposure	Medical	Pesticides	Graham and Wiener (1995); Hansen et al. (2008)
74	West Nile Virus control	Pesticide spraying can reduce the adult mosquito population and reduce the spread of West Nile Virus but increases the risk of pesticide exposure and poisoning	West Nile Virus outbreaks	Pesticide exposure	Medical	Pesticides	Hansen et al. (2008)
75	Wind power and sulfur hexafluoride (SF6)	The deployment of intermittent wind power has helped decarbonize electricity grids to address the risk of climate change but requires more of the synthetic gas SF6 for switchgear, which has potent global warming effects	Climate change	Climate change	Technological	Air	Cresswell (2022); Cody (2022)
